# Sox2 is required for olfactory pit formation and olfactory neurogenesis through BMP restriction and *Hes5* upregulation

**DOI:** 10.1242/dev.153791

**Published:** 2018-01-15

**Authors:** Tamilarasan K. Panaliappan, Walter Wittmann, Vijay K. Jidigam, Sara Mercurio, Jessica A. Bertolini, Soufien Sghari, Raj Bose, Cedric Patthey, Silvia K. Nicolis, Lena Gunhaga

**Affiliations:** 1 Umeå Centre for Molecular Medicine, Umeå University, 901 87 Umeå, Sweden; 2 Department of Biotechnology and Biosciences, University of Milano-Bicocca, 20126 Milan, Italy

**Keywords:** Sox2, Hes5, Olfactory epithelium, Neurogenesis, Mouse

## Abstract

The transcription factor Sox2 is necessary to maintain pluripotency of embryonic stem cells, and to regulate neural development. Neurogenesis in the vertebrate olfactory epithelium persists from embryonic stages through adulthood. The role *Sox2* plays for the development of the olfactory epithelium and neurogenesis within has, however, not been determined. Here, by analysing *Sox2* conditional knockout mouse embryos and chick embryos deprived of *Sox2* in the olfactory epithelium using CRISPR-Cas9, we show that Sox2 activity is crucial for the induction of the neural progenitor gene *Hes5* and for subsequent differentiation of the neuronal lineage. Our results also suggest that Sox2 activity promotes the neurogenic domain in the nasal epithelium by restricting *Bmp4* expression. The *Sox2-*deficient olfactory epithelium displays diminished cell cycle progression and proliferation, a dramatic increase in apoptosis and finally olfactory pit atrophy. Moreover, chromatin immunoprecipitation data show that Sox2 directly binds to the *Hes5* promoter in both the PNS and CNS. Taken together, our results indicate that Sox2 is essential to establish, maintain and expand the neuronal progenitor pool by suppressing *Bmp4* and upregulating *Hes5* expression.

## Abstract (precis)

**Summary:** Analysis of *Sox2* mutant mouse and Sox2 CRISPR-targeted chick embryos reveals that Sox2 controls the establishment of sensory progenitors in the olfactory epithelium by suppressing *Bmp4* and upregulating *Hes5* expression.

## INTRODUCTION

The transcription factor Sox2 belongs to the SoxB1 family (Sox1-3), which collectively are expressed in the majority, if not all, neural precursor cells in the central nervous system (CNS) (Pevny and Placzek, 2005). Moreover, Sox2 is necessary for the pluripotency of embryonic stem cells, and *Sox2* knockout mice have been shown to be early embryonic lethal (Avilion et al., 2003; Masui et al., 2007). Later in neural development, Sox2 becomes restricted to neural stem and early progenitor cells, in which it acts to maintain an undifferentiated cell state (Bylund et al., 2003; Cavallaro et al., 2008; Graham et al., 2003; Hagey and Muhr, 2014; Holmberg et al., 2008). The crucial role that Sox2 plays in self-renewal and differentiation of neural precursors has been reviewed (Maucksch et al., 2013; Pevny and Placzek, 2005; Pevny and Nicolis, 2010). In slowly dividing stem cells, high levels of Sox2 expression repress pro-proliferative genes, whereas reduced levels of Sox2 results in a transition to a proliferative progenitor cell state (Hagey and Muhr, 2014). At postnatal stages, Sox2 marks neural stem cells within the three neurogenic niches of the head region: the hippocampus, the subventricular zone (SVZ) and the olfactory epithelium (Ellis et al., 2004; Guo et al., 2010; Suh et al., 2007; Zappone et al., 2000). Several studies have examined the requirement and role of Sox2 in the CNS (reviewed by Feng and Wen, 2015; Pevny and Nicolis, 2010; Sarlak and Vincent, 2016; Shimozaki, 2014), whereas less is known about its function in the peripheral nervous system (PNS).

The olfactory epithelium, which belongs to the PNS, expresses Sox2 both during development and at adult stages (Guo et al., 2010; Krolewski et al., 2012; Pandit et al., 2011). The nasal epithelium is derived from the olfactory placode, a transient thickening of the embryonic head ectoderm in proximity to the ventral telencephalon. During development, the nasal epithelium is divided into a sensory domain and a respiratory region (Croucher and Tickle, 1989; Maier et al., 2010). The sensory epithelium produces several cell types, including olfactory sensory neurons, whereas the respiratory epithelium generates, among others, non-neural cells producing mucus that removes particles from inhaled air. The olfactory epithelium is one of few tissues, together with the hippocampus and SVZ, that maintain adult neurogenesis (Brann and Firestein, 2014; Kazanis, 2013). The role Sox2 plays in the development of the olfactory epithelium remains to be determined.

Olfactory neurogenesis begins already at the placodal stage and involves the generation of post-mitotic neurons (Fornaro et al., 2001; Maier and Gunhaga, 2009), which are among the first neurons generated in the vertebrate nervous system. During olfactory neurogenesis, distinct genes are upregulated in a sequential manner in the neuronal lineage, in the same conserved programme as for neurogenesis within the CNS. This includes *Hes5* in progenitor cells, *Ngn1* (also called *Neurog1*) in immediate neuronal precursor cells, *NeuroD* (*Neurod1*) in cells committed to leave the cell cycle, and *HuC/D* (*Elavl3/4*) and class III β-Tubulin (*Tubb3*) in post-mitotic neurons (Cau et al., 2002, 2000; Fornaro et al., 2003; Maier and Gunhaga, 2009; Wei et al., 2013; Wittmann et al., 2014a,b). However, *Ngn1* expression is maintained in both differentiated *Neurod1* cells and post-mitotic neurons before being downregulated (Maier and Gunhaga, 2009). The roles of distinct transcription factors necessary for cell cycle exit, downregulation of progenitor proteins and upregulation of neuron differentiation markers have been well characterized (reviewed by Bertrand et al., 2002; Kam et al., 2014; Ross et al., 2003; Urban and Guillemot, 2014). Neurogenesis has been shown to involve similar molecular mechanisms at embryonic and adult stages, both in the olfactory epithelium and in the brain, across several vertebrate species (Bonaguidi et al., 2008; Kohl et al., 2010; Lazic et al., 2004; Maier et al., 2011). Thus, the relatively simple and easily accessible olfactory epithelium provides a good model system for studying the interactions of signalling molecules and downstream transcription factors, and how they act during neurogenesis (Cau et al., 1997; Fletcher et al., 2011; Kam et al., 2016; Kawauchi et al., 2009; Maier et al., 2011; Packard et al., 2011; Tucker et al., 2010; Wittmann et al., 2014a). The function of Sox2 in neurogenesis in the olfactory epithelium has not yet been addressed.

In this study, we have analysed the role of *Sox2* in the development of the olfactory epithelium and neurogenesis within. To examine this, we used a conditional *Foxg1-* (previously known as *Bf1*) *Cre* mouse line to delete *Sox2* in the olfactory placode. We also disrupted *Sox2* in the developing chick olfactory epithelium by designing a CRISPR-*Sox2* vector and using the CRISPR/Cas9 system. Our results show that *Sox2* deficiency results in upregulation of *Bmp4* expression, disruption of olfactory epithelium development, including loss of the early neurogenic marker *Hes5*, diminished cell cycle progression and proliferation, and complete depletion of the neuronal lineage. Moreover, we also detected increased apoptosis and finally olfactory pit atrophy. Our data further show that mutations in Sox2-binding sites of the *Hes5* promoter result in loss of cis-regulatory activity. Taken together, our findings suggest that Sox2 promotes the olfactory sensory domain by repressing BMP activity, and acts as a regulator of *Hes5* expression and the subsequent onset of neurogenesis.

## RESULTS

### *Sox2* expression becomes progressively restricted to the sensory part of the olfactory epithelium

**Fig. 1.**
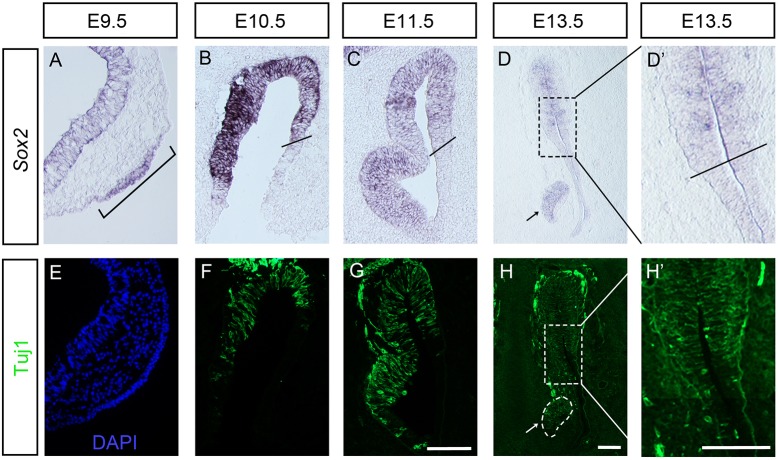
***Sox2* and Tuj1 expression patterns in the olfactory epithelium of wild-type mouse.** (A) At E9.5, expression of *Sox2* is detected in the olfactory placode (bracket). (B-D′) From E10.5, *Sox2* expression is restricted to the olfactory sensory epithelium. Black lines in B, C and D′ indicate the border between the sensory and respiratory epithelia. (E) At E9.5, no or only a few Tuj1^+^ neurons are detected in the olfactory placode. (F-H′) From E10.5, Tuj1^+^ neurons are located in the medial part of the sensory epithelium. (D,H) *Sox2* and Tuj1 are also expressed in the vomeronasal organ, an olfactory epithelial derivative (indicated by arrows). D′ and H′ show higher magnifications of the boxed areas in D and H. Scale bars: 100 µm.

First, we examined the expression of *Sox2* in the early forming olfactory epithelium in mouse embryos. At embryonic day (E) 9.5, the olfactory placode becomes morphologically visible as an epithelial thickening of the head ectoderm near the telencephalon (Fig. 1A). Already at this early stage, olfactory placodal cells express *Sox2* (Fig. 1A), and we have previously shown that cells of the neuronal lineage including a few post-mitotic neurons are detectable in the newly formed placode (Wittmann et al., 2014a). From E10.5, neurogenesis is located in the medial sensory part of the olfactory epithelium (Fig. 1B-D,F-H) (Kawauchi et al., 2009; Wittmann et al., 2014a). Consistently, at these stages, *Sox2* expression is restricted to the sensory olfactory epithelium, whereas the respiratory part of the olfactory epithelium is *Sox2* negative (Fig. 1B-D). We observed the strongest expression of *Sox2* at E10.5, and lower levels at later stages (Fig. 1A-D).

### Loss of Sox2 inhibits the neuronal lineage in the olfactory placode in mouse

**Fig. 2.**
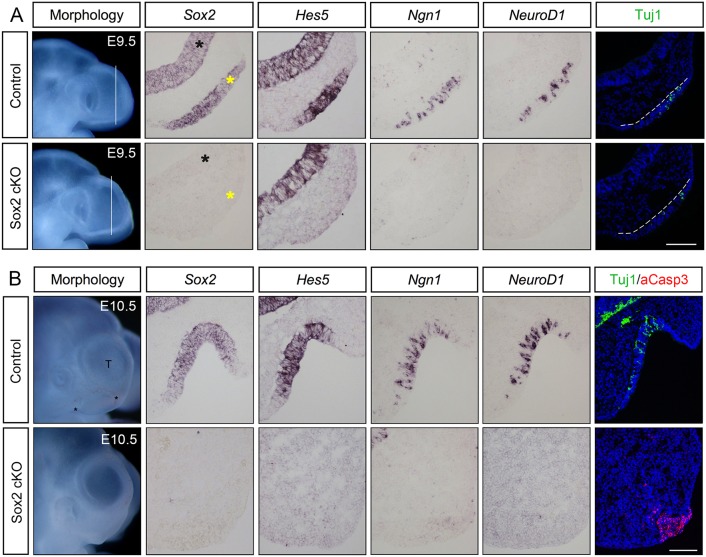
**Loss of neurogenic markers in the *Sox2*-deficient olfactory epithelium.** (A) At E9.5, cells in the control olfactory placode express *Sox2. Hes5*^+^ stem-like progenitors, *Ngn1*^+^ neuronal precursors, *Neurod1*^+^ and Tuj1^+^ post-mitotic neurons are detected in the olfactory placode (*n*=5). In E9.5 *Sox2*-deficient olfactory placodes, no expression of *Sox2*, *Hes5, Ngn1* or *Neurod1*, and a reduced number of Tuj1^+^ cells are detected (*n*=4). The telencephalon is indicated by black asterisks, whereas the olfactory placode is marked by yellow asterisks, as well as by broken white lines. (B) At E10.5, the control olfactory epithelium is invaginated into a pit-like structure and sensory cells express *Sox2*; *Hes5*^+^ stem-like progenitors, *Ngn1*^+^ neuronal precursors, *Neurod1*^+^ and Tuj1^+^ post-mitotic neurons are detected in the epithelium (*n*=6). No or very few aCaspase3^+^ apoptotic cells are detected in the control olfactory epithelium (*n*=6). By E10.5, *Sox2* cKO mutants (*n*=4) do not exhibit an olfactory epithelium, and no expression of any neurogenic markers is detected. A cluster of aCaspase3^+^ apoptotic cells is observed within the area of the disrupted olfactory epithelium. Scale bars: 100 µm.

To explore the role of Sox2 during early development of the olfactory epithelium and neurogenesis within, we first analysed *Sox2*-deficient embryos and their control littermates. The *Sox2* transgenic mice were generated by breeding mice carrying a *Sox2^flox^* conditional mutation (Favaro et al., 2009) with mice expressing the Cre-recombinase gene under the control of the *Foxg1* promoter (Hébert and McConnell, 2000). The *Sox2^flox/flox^*; *FoxG1^Cre/+^* embryos (Ferri et al., 2013) are hereafter referred to as *Sox2* conditional knockout (cKO) embryos. The expression of *Foxg1* is detected already at the initiation of neurogenesis in the olfactory placode of mice (Xuan et al., 1995), and also at later stages of olfactory development (Kawauchi et al., 2009). At E9.5, *Sox2* is normally expressed throughout the olfactory placode (Fig. 2A). In E9.5 *Sox2* cKO embryos, Cre-mediated deletion of *Sox2* had already occurred in the olfactory placode as well as the telencephalon, but without any clear morphological disturbances of the olfactory placode (Fig. 2A). In addition, the olfactory placode markers *Dlx3* and *Dlx5* (Bhattacharyya and Bronner-Fraser, 2008) were expressed throughout the placode in both wild-type and Sox2 cKO embryos at E9.5 (Fig. S1), suggesting that Sox2 is not required for the initial formation of the olfactory placode.

**Fig. 3.**
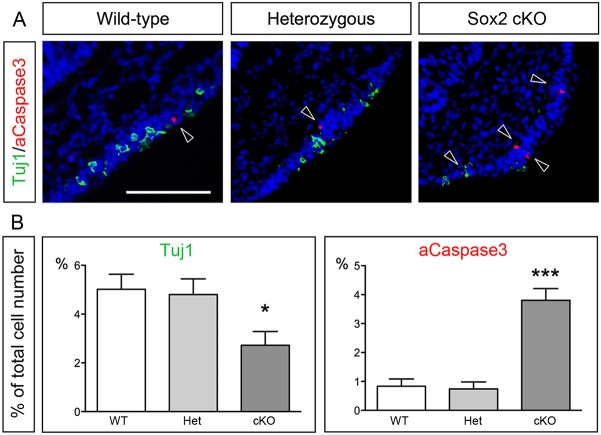
**Decreased neuronal differentiation and increased apoptosis in the *Sox2*-deficient olfactory epithelium.** (A) Tuj1 and aCaspase3 immunofluorescence in E9.5 wild-type, heterozygous *Sox2^flox/+^* and *Sox2* cKO mutants. Arrowheads indicate aCaspase3^+^ cells. (B) No change in the number of Tuj1^+^ neurons or aCaspase^+^ apoptotic cells between the wild-type and heterozygous mice are detected. In contrast, the generation of Tuj1^+^ neurons is decreased and aCaspase3^+^ apoptotic cells are increased in *Sox2* cKO mutants compared with wild-type and heterozygous mice. Statistical analysis of the cell counts in comparison to the total cell number in the olfactory epithelium of E9.5 wild-type (*n*=7), heterozygous (*n*=6) and *Sox2* cKO mutants (*n*=5) are as follows. For Tuj1, wild type versus Het *P*=0.8173, wild type versus KO *P*=0.0263, Het versus KO *P*=0.0412. For aCaspase3, wild type versus Het *P*=0.7847, wild type versus KO *P*<0.0001, Het versus KO *P*<0.0001. Error bars represent s.e.m. Student's *t*-test, *P<0.05, ***P<0.0001. Scale bar: 100 µm.

Next, we analysed whether early stages of olfactory neurogenesis were affected in *Sox2* cKO embryos. At E9.5, the entire neuronal lineage was abolished, with a complete lack of *Hes5^+^* progenitors, *Ngn1^+^* neural precursors and *Neurod1^+^* differentiated neurons in the olfactory placode of *Sox2* cKO mutants, in contrast to control littermates (Fig. 2A). Only a few Tuj1^+^ post mitotic neurons were detectable in the mutant olfactory placode (Fig. 2A), suggesting a short period of intact neurogenesis before the Cre-mediated *Sox2* ablation occurred. There was no difference in the generation of *Hes5^+^*, *Ngn1^+^*, *Neurod1^+^* or Tuj1^+^ cells between wild-type embryos and heterozygous *Sox2^flox/+^* mutants (Fig. 3 and data not shown). In addition, *Hes5* was still expressed in the forebrain of *Sox2* cKO mutants (Fig. 2A), suggesting that Sox2-mediated regulation of *Hes5* expression is context dependent. Taken together, E9.5 *Sox2* cKO embryos exhibit a loss of cells at various stages of neuronal differentiation in the olfactory placode.

### The *Sox2*-deficient olfactory placode exhibits changes in cell cycle progression, proliferation and cell death

Next, we examined whether changes in proliferation and/or cell death could explain the loss of neurogenic cell types in the Sox2-deficient embryos at E9.5. The number of apoptotic cells, defined by activated (a) Caspase 3, was significantly increased in the olfactory placode compared with wild-type embryos (Fig. 3A,B). In contrast, the number of aCaspase3^+^ apoptotic cells was identical between wild-type and heterozygous *Sox2^flox/+^* littermates (Fig. 3A,B). Notably, apoptotic Tuj1^+^ neurons were never detected in any of the genotypes (Fig. 3A).

To determine the proliferation rate and the progression of the cell cycle in *Sox2* cKO mutants, E9.5 embryos were pulsed for 2 h with BrdU, and for 30 min with EdU starting 1.5 h after the initiation of the BrdU pulse, before being sacrificed. The number of EdU^+^ cells in S-phase in *Sox2* cKO mutants was significantly reduced compared with wild-type and heterozygous *Sox2^flox/+^* embryos (Table 1). In addition, the length of the S-phase (Ts) and the total cell cycle length (Tc) were significantly longer in *Sox2* cKO mutants compared with wild-type littermates (Table 1). Taken together, one intact *Sox2* allele is sufficient for initial neurogenesis and proliferation in the olfactory placode. In contrast, homozygous *Sox2* deficiency severely affects the developing olfactory epithelium, as shown by diminished neurogenesis, increased apoptosis, reduced proliferation and slower progression of the cell cycle.

**Table 1.**
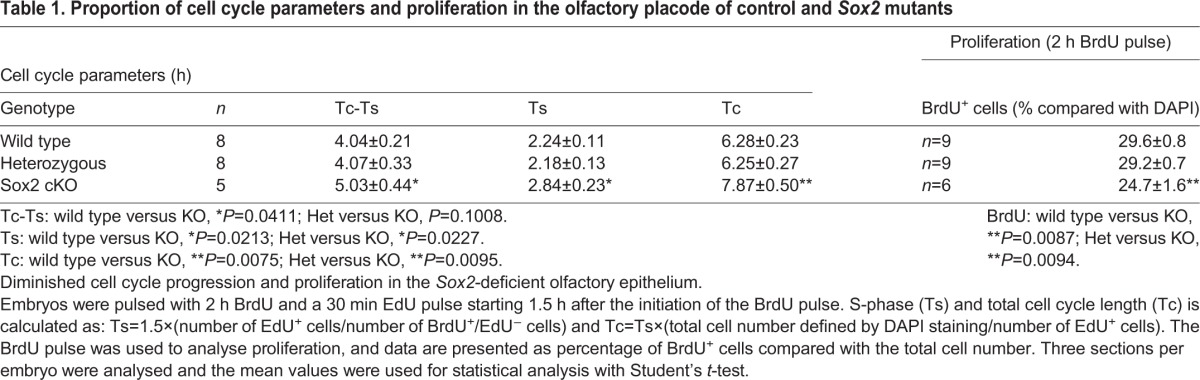
**Proportion of cell cycle parameters and proliferation in the olfactory placode of control and *Sox2* mutants**

### The olfactory pit fails to form in Sox2-deficient embryos at E10.5

To further investigate the effect of *Sox2* deficiency on the development of the olfactory epithelium, *Sox2* cKO mutants and control littermates were analysed at E10.5. In wild-type embryos, the olfactory placode begins to invaginate around E10 and forms the olfactory pit. At E10.5, the olfactory pit is clearly detectable as a depression of the head ectoderm at the most anterior-ventral part of the telencephalon (Figs 1B and 2B). At this stage, *Sox2* expression is restricted to the neuronal sensory part of the olfactory epithelium in wild-type embryos, where the neurogenic markers *Hes5*, *Ngn1*, *Neurod1* and Tuj1 also are expressed (Fig. 2B). No aCaspase3^+^ apoptotic cells were detected in the olfactory epithelium of control littermates (Fig. 2B).

Strikingly, *Sox2* cKO mutant embryos did not show any morphological structure of the olfactory epithelium at E10.5, correlating with expression of *Sox2* being completely absent in the anterior-ventral part of the head region (Fig. 2B). Moreover, no *Hes5^+^* stem-like progenitors, *Ngn1^+^* neural precursors, *Neurod1^+^* differentiated neurons or Tuj1^+^ post-mitotic neurons were detected in the anterior-ventral part of the head region (Fig. 2B). Consistent with the observed olfactory pit atrophy, there was a large cluster of aCaspase3^+^ apoptotic cells localized within the area of the disrupted olfactory epithelium (Fig. 2B). Thus, at E10.5, *Sox2*-deficient mice completely lack an olfactory epithelium, including any cells of the neuronal lineage, which in part is explained by massive apoptosis in the olfactory epithelial domain.

### Sox2 inhibition leads to cell-autonomous downregulation of neurogenic markers and upregulation of respiratory markers

To examine a direct role of Sox2 in neurogenesis, without possible effects caused by severe morphological disruption of the olfactory epithelium, we turned to chick as a model system. *In ovo* chick electroporation assays have the advantage that specific domains of interest in the embryo can be targeted by gene constructs, which lead us to design an electroporation construct to disrupt the *Sox2* gene by using the CRISPR/Cas9-system (Hille and Charpentier, 2016). The onset of neurogenesis in the chick olfactory placode is around stage 14 (Fornaro et al., 2003; Maier and Gunhaga, 2009). Therefore we electroporated stage 9/10 chick embryos (∼E8.5 in mouse) in the prospective olfactory region with a control vector expressing GFP (Yaneza et al., 2002) alone or together with a *Sox2* guide (g) RNA-expressing vector (*Sox2*-CRISPR) and the h*Cas9* vector (the combination of the two latter vectors is herein called *Sox2*-CRISPR/Cas9). A scrambled gRNA construct (*Cont*-CRISPR) together with the *GFP* and h*Cas9* vectors was used as a control. The electroporated embryos were cultured to approximately stage 20-22, and embryos with GFP staining within the olfactory region were collected for analyses.

**Fig. 4.**
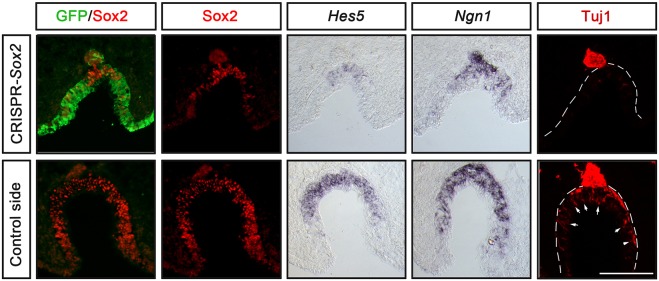
**Inhibition of Sox2 in the chick olfactory epithelium downregulates neurogenic markers in a cell-autonomous manner.** *In ovo* electroporation of stage 9/10 chick embryos in the olfactory epithelium using pCAβ-*EGFP*-m5 and *Sox2-*CRISPR/Cas9 constructs, cultured to approximately stage 20-22. The *Sox2-*CRISPR/Cas9 electroporated olfactory pit is smaller compared with the control non-electroporated side. The generation of *Hes5^+^* and *Ngn1^+^* cells, and the number of Tuj1^+^ neurons are reduced in the regions of disrupted Sox2 expression in the olfactory epithelium compared with the control side (*n*=7). Tuj1^+^ neurons in the olfactory epithelium (outlined) of the control side are indicated with arrows. A reduction in the number of migratory Tuj1^+^ neurons is also detected outside of the olfactory epithelium (outlined) in *Sox2-*CRISPR/Cas9 electroporated embryos. Scale bar: 100 µm.

All embryos electroporated with the *GFP* control vector alone or together with the *Cont-*CRISPR vector displayed normal morphology of the olfactory epithelium, and the expression of *Sox2*, *Hes5* and *Ngn1*, and the numbers of Tuj1^+^ post-mitotic neurons were unchanged compared with the non-electroporated side (Fig. S2). In contrast, in *Sox2*-CRISPR-electroporated embryos Sox2 expression was eliminated in targeted GFP^+^ cells in the olfactory epithelium (Fig. 4). In addition, the generation of *Hes5^+^* and *Ngn1^+^* cells and Tuj1^+^ neurons were reduced in the regions of disrupted Sox2 expression in the olfactory epithelium (Fig. 4). Consistently, the number of migratory Tuj1^+^ neurons, emanating from the olfactory epithelium (Fornaro et al., 2003; Maier and Gunhaga, 2009; Maier et al., 2011), was also reduced (Fig. 4). The olfactory pit was also smaller in size in embryos with electroporation efficiency that was more than 50% of the epithelium (Fig. 4). In GFP-negative regions expression of Sox2 could still be detected, together with weak expression of *Hes5* and *Ngn1*, pointing to a cell-autonomous effect (Fig. 4). Thus, disruption of *Sox2* activity in the chick olfactory epithelium leads to disrupted olfactory neurogenesis. Taken together, the data from mice and chick suggest that the requirement for Sox2 in olfactory neurogenic development is conserved in different vertebrate lineages.

### *Hes5* expression is the earliest indication of neural determination in the olfactory epithelium

In relation to sensory versus respiratory patterning of the olfactory epithelium, studies in chick have shown that *Hes5* is expressed prior to other neuronal markers in the olfactory epithelium (Maier and Gunhaga, 2009). We therefore examined the expression pattern of neuronal markers in mouse at E9.0. Consistent with the chick data, *Hes5* is already expressed in the olfactory placodal region at E9.0 in mouse, prior to *Ascl1* (previously *Mash1*), *Ngn1*, *Neurod1* and Tuj1 expression (Fig. S3). These results indicate that *Hes5* might be the earliest marker associated with neuronal determination in the sensory olfactory epithelium.

Hes genes are also known to be downstream targets of Notch signalling (reviewed by Iso et al., 2003; Kageyama and Ohtsuka, 1999), raising the possibility that Sox2 promotes *Hes5* expression indirectly through changes in Notch signalling. However, expression of *Notch1* was not detected prior to E10.5 in the olfactory placode of wild-type embryos, and expression of *Notch1* and *Delta1* were unchanged in *Sox2* cKO embryos compared with wild-type littermates (Fig. S4). These results suggest that the initial expression of *Hes5* in the olfactory placode is dependent on Sox2, but not on Notch activity.

### *Sox2*-binding sites in the *Hes5* promoter are crucial for cis-regulatory activity

As the entire neuronal lineage is depleted in the *Sox2*-deficient embryos (Figs 2 and 4) and *Hes5* is one of the earliest markers of the neuronal lineage (Cau et al., 2000; Ohtsuka et al., 1999), we investigated the possibility that Sox2 directly activates *Hes5* expression. Motif searches in the *Hes5* upstream sequence identified one predicted Sox2-binding site located at position −504 bp upstream of the transcription start site and four additional sites between positions −212 and −99. These four sites were conserved between species, as shown by alignment of the mouse, human, chick and ostrich homologous sequences (Fig. S5). In addition, mining of six publicly available ChIP-Seq data sets revealed a peak of Sox2 binding centred to this region of the *Hes5* promoter in human and mouse cells (Fig. S6). These data sets included: mouse embryonic cortex and spinal cord neural progenitors; transformed otic progenitors; forebrain-like ES cell-derived neural progenitors; a human neural stem cell line of ventral midbrain origin; and forebrain-like ES cell-derived neural progenitors (Hagey et al., 2016; Kwan et al., 2015; Lodato et al., 2013; Ng et al., 2013; Sancho-Martinez et al., 2016). Together, these data show that Sox2 binds to the *Hes5* promoter region in neural progenitors in both the CNS and PNS.

**Fig. 5.**
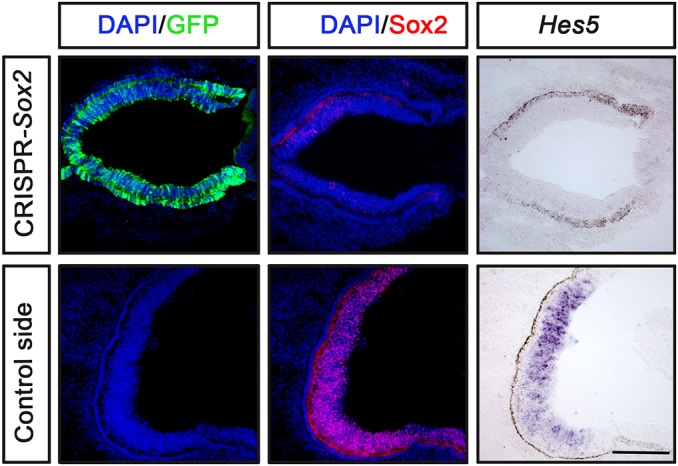
**Inhibition of Sox2 in the chick retina downregulates *Hes5*.** *In ovo* electroporation of stage 10 chick embryos in the optic vesicle using pCAβ-*EGFP*-m5 and *Sox2-*CRISPR/Cas9 constructs, and cultured to approximately stage 24 (*n*=4). The generation of *Hes5^+^* cells is reduced in the regions of disrupted Sox2 expression in the retina compared with the control side. The *Sox2-*CRISPR/Cas9 electroporated retina is smaller compared with the control non-electroporated side. Scale bar: 100 µm.

To further examine whether *Hes5* expression is dependent on Sox2 activity in the CNS, the *Sox2*-CRISPR/Cas9 and GFP constructs were electroporated in the retina at stage 10 and cultured to ∼E4. Under these conditions, the *Hes5* expression in the retina was reduced or completely inhibited (Fig. 5). Together, these results suggest that *in vivo* expression of *Hes5* is regulated by Sox2 in regions of both the CNS and PNS.

**Fig. 6.**
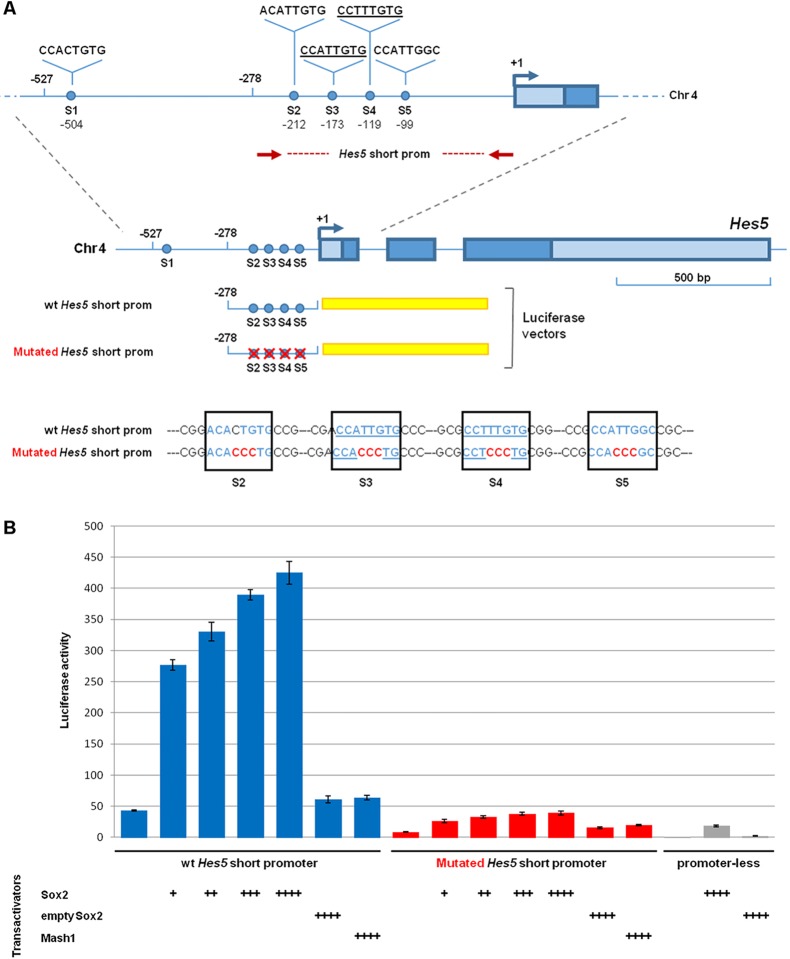
**Cis-regulatory activity of the *Hes5* promoter is disrupted by mutations in predicted Sox2-binding sites.** (A) The *Hes5* promoter region. The red dotted line indicates the position of the short *Hes5* promoter region cloned in a luciferase vector. Putative Sox2-binding sites (S1 to S5) are indicated by blue dots. The underlined sequences are the most confident Sox2 consensus motifs. A comparison of the S2- to S5-binding sites in the cloned wild-type and mutated *Hes5* promoter fragment is shown at the bottom. (B) Promoter activation assay in Neuro2a cells transfected with a wild-type (wt; blue bars) or mutated (red bars) *Hes5* short promoter cloned in a luciferase vector. Co-transfection of increasing amounts of *Sox2*-expressing vector, but not of a *Mash1*-expressing vector or a control empty vector, resulted in a dose-dependent increase in luciferase activity driven by the wild-type *Hes5* promoter (blue bars), but not by the mutated Hes5 promoter (red bars). *Sox2-* or *Mash1-*expressing vectors did not induce luciferase activity in co-transfection with a promoter-less luciferase vector. The molar ratios compared with the luciferase vector (set at 1) were: +, 1:0.075; ++, 1:0.125; +++, 1:0.25; ++++, 1:0.5. Results are represented as fold-change increase in activity compared with the promoter-less luciferase vector without co-transfected Sox2, which is set at 1. Values are the mean of three independent transfection experiments carried out in triplicate. Error bars represent s.e.m.

To assess whether Sox2 is sufficient for cis-regulatory activation of the *Hes5* promoter, a 527 bp DNA fragment (herein named long promoter) or a 278 bp fragment (herein named short promoter) of the mouse *Hes5* promoter were fused to DNA encoding luciferase (Mariani et al., 2012) (Fig. 6A and Fig. S7A). These *Hes5-luciferase* constructs were transfected in Neuro2a cells alone or together with increasing amounts of a *Sox2* expression construct (Favaro et al., 2009) (Fig. 6 and Fig. S7). Although a control Sox2-empty vector had no effect on luciferase activity, co-transfection of increasing amounts of a Sox2-expressing vector led to a significant, dose-dependent transactivation of both long and short Hes5 promoter fragments (Fig. 6 and Fig. S7). As the short promoter corresponded exactly to the peak of Sox2 binding in the ChIP-Seq data sets (Fig. S6), we focused on this region for further investigations. To evaluate a direct action of Sox2 on *Hes5* regulation, all four conserved *Sox2*-binding sites within the short promoter were mutated. Importantly, these mutations abolished the Sox2-dependent induction of *Hes5* promoter activity (Fig. 6B), indicating that Sox2 is a direct regulator of *Hes5* expression. Co-transfection with a control vector expressing the neurogenic determinant *Ascl1*, instead of *Sox2*, did not have any effect (Fig. 6B), pointing to the Sox2 specificity of the observed activation response.

### The *Hes5* promoter is activated in olfactory epithelial cells *in vivo*

To further determine whether the *Hes5* promoter can be activated in olfactory epithelial cells, we cloned the short *Hes5* promoter to drive GFP expression (Fig. S8). Subsequently, this construct was electroporated *in ovo* in the olfactory placodal region around stage 10 and embryos were cultured to approximately stage 20-22. As a control the Sox2-negative caudal dorsal ectoderm was electroporated with the *Hes5-*GFP construct. During these conditions, GFP expression was detected in the olfactory epithelium, but not in caudal dorsal ectodermal cells (Fig. S8), providing evidence that the *Hes5* promoter can be activated in olfactory epithelial cells *in vivo*.

### Ectopic *Hes5* expression is not sufficient to induce neurogenic character in respiratory epithelium

To examine whether *Hes5* has the potential to ectopically induce neurogenesis in prospective respiratory cells or the head ectoderm near the olfactory region, we continued to take advantage of the chick model system. A GFP-construct was electroporated together with a *Hes5-*overexpression construct (Holmberg et al., 2008) in the respiratory ectoderm and head ectoderm near the olfactory region in stage 10/11 embryos. The electroporated embryos were cultured to approximately stage 22 and embryos with GFP staining within the olfactory regions were collected for analyses. All embryos electroporated exhibited a normal morphology of the olfactory region, and ectopic *Hes5* activity in the respiratory domain was not sufficient to inhibit *Msx1/2* expression or to induce neurogenesis, which are marked by *Ngn1* and Tuj1 in comparison with the non-electroporated control side (Fig. S9). Thus, our results suggest that ectopic *Hes5* expression is not sufficient to trigger neurogenesis in the respiratory epithelium.

### Sox2 restricts the respiratory domain and promotes the neuronal lineage independently of BMP activity

The suppression of the neuronal lineage in the Sox2-deficient olfactory epithelium might be due to an early patterning defect. It is known that during early development, the olfactory epithelium becomes restricted into an anterior-medial sensory region and a posterior-lateral respiratory domain (Croucher and Tickle, 1989; Maier et al., 2010). This division of the nasal epithelium is regulated by FGF and BMP signals that promote sensory and respiratory cell identities, respectively (Maier et al., 2010). Therefore, we analysed the expression of *Fgf8*, *Bmp4* and the olfactory respiratory marker *Msx2* (Maier et al., 2010) in E9.5 and E10.5 *Sox2* cKO mutants and control littermates.

**Fig. 7.**
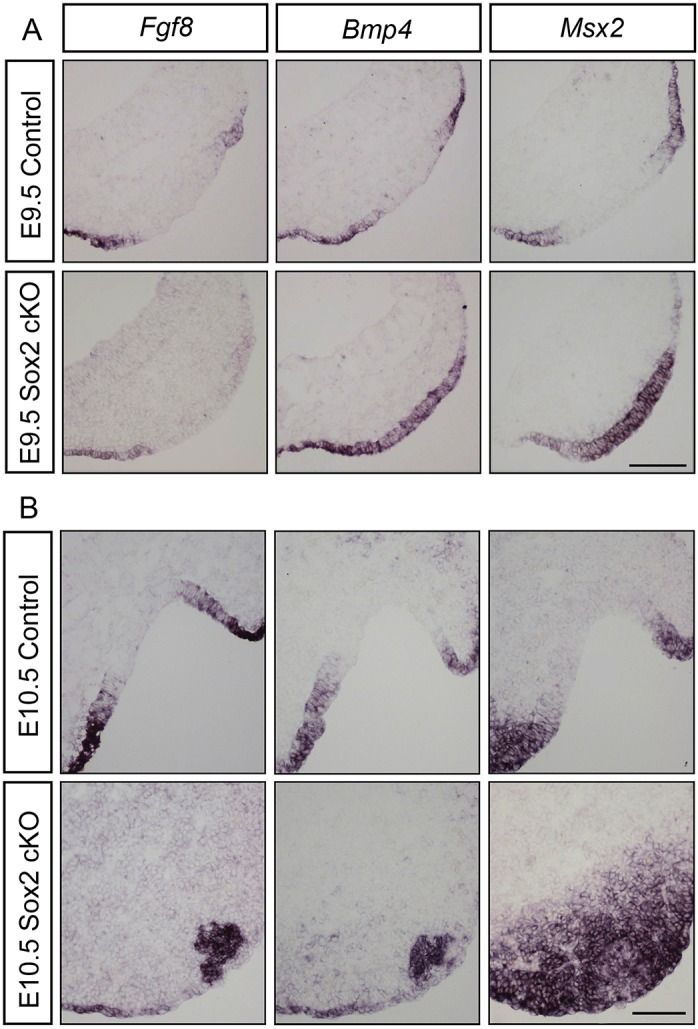
**Increased *Bmp4* and *Msx2* expression in the Sox2-deficient olfactory sensory epithelium.** (A) At E9.5, in control embryos (*n*=5), *Fgf8*, *Bmp4* and *Msx2* expression are overlapping at the edges of the olfactory placode, the prospective respiratory region. In *Sox2* cKO mutants (*n*=4), *Bmp4* and *Msx2* expression is expanded into the entire olfactory placode, and *Fgf8* expression is mildly decreased at the edges of the placode. (B) At E10.5, in the centre of the olfactory pit of control embryos (*n*=5), *Fgf8*, *Bmp4* and *Msx2* expression overlap in the lateral edges of the epithelium. In E10.5 *Sox2* cKO mutants (*n*=4), *Fgf8*, *Bmp4* and *Msx2* are co-expressed in a small cluster in the anterior-ventral part of the head region, and *Msx2* is also expressed in the adjacent mesenchyme and surface ectoderm. Scale bars: 100 µm.

At E9.5, *Fgf8*, *Bmp4* and the respiratory marker *Msx2* were expressed in an overlapping pattern in the lateral parts of the olfactory placode in control embryos (Fig. 7A). In addition, in E10.5 control embryos, at the centre of the olfactory pit, *Fgf8* and *Bmp4* expression overlaps in the lateral edges of the epithelium where *Msx2* expression is also detected (Fig. 7B). In contrast, in *Sox2* cKO mutants, *Fgf8* expression was mildly decreased, but still restricted to the edges of the placode, whereas *Bmp4* and *Msx2* expression were expanded to the entire olfactory placode (Fig. 7A), which is consistent with the finding that BMP signals induce *Msx2*^+^ respiratory cells (Maier et al., 2010). Moreover, in E10.5 *Sox2* cKO mutants, *Fgf8*, *Bmp4* and *Msx2* were co-expressed in a small area in the anterior-ventral part of the head region (Fig. 7B) corresponding to the aCaspase3-rich domain (Fig. 2B). In addition, in the *Sox2*-deficient embryos, *Msx2* was expressed throughout the surface ectoderm without any interruptions and in the adjacent mesenchyme (Fig. 7B). These results indicate that *Sox2* acts as a negative regulator of *Bmp4* expression to restrict the respiratory domain and favour olfactory sensory cell identity in the nasal epithelium.

**Fig. 8.**
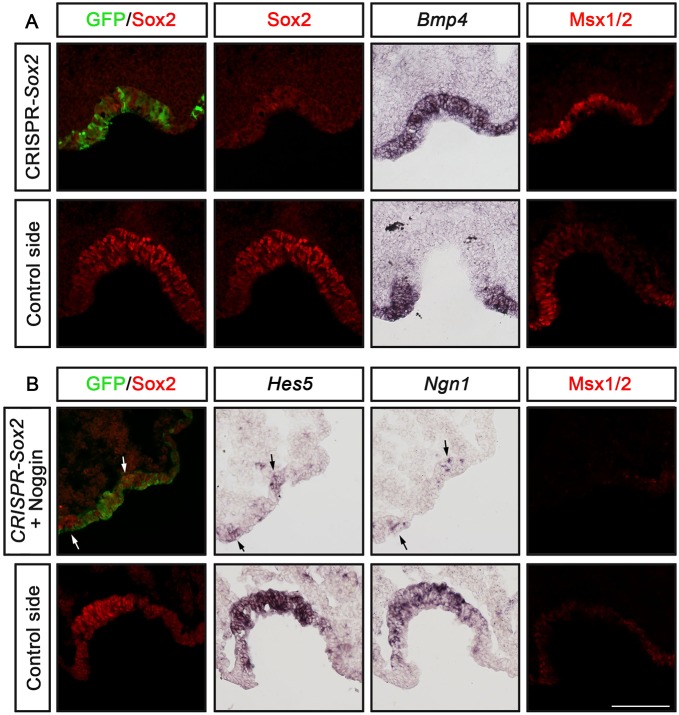
***Bmp4* expression is upregulated in Sox2-deficient cells, but in the absence of BMP activity Sox2 is still required for the neurogenic lineage.** (A,B) *In ovo* electroporation of stage 9/10 chick embryos in the olfactory epithelium using pCAβ-*EGFP*-m5 and *Sox2-*CRISPR/Cas9 constructs alone (A; *n*=5) or together with a Noggin vector (B; *n*=4), and cultured to approximately stage 20-22. The electroporated olfactory pit is smaller compared with the control non-electroporated side. (A) The expression of *Bmp4* and Msx1/2 is expanded in the Sox2-deficient region of the nasal epithelium. (B) In regions of suppressed Sox2 and BMP activity, observed by loss of Sox2 and Msx1/2 expression, respectively, *Hes5* and *Ngn1* expression is reduced. A few *Hes5^+^* and *Ngn1^+^* cells are detected in regions with remaining Sox2 activity (arrows). Scale bar: 100 µm.

We further examined this issue using *Sox2*-CRISPR-electroporations in the prospective olfactory region in chick embryos. In Sox2-CRISPR-electroporated embryos, *Bmp4* and Msx1/2 expression expanded into the Sox2 deficient region of the nasal epithelium, which in some cases also were less invaginated (Fig. 8A). To test whether inhibition of neurogenesis is merely a result of the upregulated BMP activity, we electroporated Sox2-CRISPR/Cas9 together with a Noggin construct, which inhibits BMP signals (Timmer et al., 2002). Under these conditions, the olfactory pit formation was clearly disrupted (Fig. 8B), which is in agreement with our previous publications showing that BMP activity is regulating epithelial invagination (Jidigam et al., 2015). Moreover, the expression of Msx1/2 was suppressed in electroporated cells, and Sox2-deficient cells did not express *Hes5* and *Ngn1* (Fig. 8B), suggesting that Sox2 is required for the neurogenic lineage in the absence of BMP activity. Taken together, our results show that Sox2 activity plays a crucial role in the generation of the neurogenic lineage in the nasal epithelium, in part by restricting BMP activity and thereby promoting the sensory domain, and by regulating the onset of *Hes5* expression.

## DISCUSSION

Here, we present evidence that Sox2 activity plays a crucial role in the early development of the neural domain of the olfactory epithelium and neurogenesis within, in part by restricting *Bmp4* expression and respiratory cell identity. Our data show that mutations of the Sox2-binding sites in the *Hes5* promoter suppress cis-regulatory activity, suggesting that Sox2 might regulate neurogenesis cell-autonomously via direct activation of the *Hes5* gene. Consistently, blockade of Sox2 activity in the olfactory domain results in a complete depletion of the entire neuronal lineage. Furthermore, our results show that the *Sox2-*deficient olfactory epithelium displays diminished cell cycle progression and proliferation, a dramatic increase in apoptosis and finally olfactory pit atrophy.

By analysing *Sox2* conditional knockout mice, our results show that in the absence of *Sox2*, cells of the neuronal lineage in the olfactory epithelium already fail to develop at E9.5, the placode stage. This lack of neurogenesis was not caused by a delay in the generation of neural progenitors, because, also at later stages, no *Hes5*^+^ neural progenitor cells could be observed. Moreover, the complete inhibition of neural progenitors and precursors with only a few post-mitotic neurons detected at E9.5 provides evidence that neural progenitors do not differentiate prematurely. Despite the absence of the neuronal lineage, at this stage the morphology of the placode appeared normal and cells expressed the placode markers *Dlx3* and *Dlx5* (Bhattacharyya and Bronner-Fraser, 2008). Sox2 has also been shown to be crucial for neurosensory precursor formation in the otic placode, giving rise to the inner ear (Dabdoub et al., 2008; Kiernan et al., 2005; Neves et al., 2011, 2012). Moreover, in chick, the use of a dominant repressor *Sox3* construct, another SoxB1 family member, blocks neurogenesis in the epibranchial placodes (Tripathi et al., 2009). Thus, Sox2 and other SoxB1 family members appear to be crucial for embryonic neurogenesis in the placodes.

Besides the finding of suppressed neurogenesis, our results indicate a drastic increase of apoptotic cell death in the Sox2-deficient olfactory epithelium in mouse. These data suggest that Sox2 might play a role in regulating apoptosis. Consistently, previous studies have shown an increased apoptotic cell death in both the ventral telencephalon and in the hippocampus in *Sox2* conditional knockout mouse (Favaro et al., 2009; Ferri et al., 2013). Moreover, recent studies using *in vitro* and *in vivo* assays of neural stem cell and human lung cancer cell survival have demonstrated that silencing of *Sox2* results in decreased levels of survivin, a member of the inhibitor of apoptosis protein family, and induced apoptosis (Chen et al., 2014; Feng et al., 2013). Further studies regarding the potential role for *Sox2* in protecting neural stem and progenitor cells from apoptosis will be interesting to follow.

The nasal epithelium is divided into a neurogenic sensory region and a non-neurogenic respiratory domain (Croucher and Tickle, 1989; Maier et al., 2010). Our previous study has shown that BMP signals promote the specification of respiratory epithelial cells in the nasal epithelium, and that Msx1/2 and *Id3* can be used to detect respiratory epithelial cells (Maier et al., 2010). Furthermore, the same study provided evidence that BMP and FGF signals act in an opposing manner to regulate the respiratory versus sensory epithelial cell fate decision (Maier et al., 2010). Our results now show that in the absence of Sox2 activity, *Bmp4* and *Msx2* expression are expanded in the Sox2-disrupted olfactory epithelial domain. These results indicate that Sox2 acts as a negative regulator for *Bmp4* expression to restrict the respiratory domain and define the sensory domain of the nasal epithelium. However, inhibition of BMP activity in the Sox2-deficient olfactory cells, could not rescue neurogenesis, indicating a direct requirement for Sox2 in establishing the neuronal lineage in the olfactory epithelium. Our data also show that ectopic *Hes5* activity is not sufficient to inhibit respiratory cell identity or induce neurogenic properties in the respiratory domain, further indicating that Sox2 plays a crucial role in the establishment of the olfactory sensory territory. Future studies are required to fully understand the epistasis and hierarchy between Sox2, Hes5, Bmp4 and respiratory markers such as Msx2, and to provide deeper knowledge regarding the early development of the sensory and non-sensory nasal epithelium.

Our results indicate that in the absence of *Sox2,* the proliferative sensory progenitor pool in the olfactory epithelium is not established, resulting in complete loss of the neuronal lineage in the olfactory epithelium. Our data in chick provide evidence that inhibition of Sox2 leads to downregulation of neuronal markers in a cell-autonomous manner. Moreover, the *Sox2-*deficient olfactory epithelium exhibit reduced proliferation and a slower progression of the cell cycle. This correlates well with previous work in the olfactory epithelium that has shown that rapid proliferation occurs in the medial neurogenic part of the olfactory epithelium, in which Sox2 is highly expressed together with *Hes5* and *Mash1* (Tucker et al., 2010; Wittmann et al., 2014b). In contrast, proliferation in the lateral part of the olfactory epithelium proceeds in a slow and symmetric manner (Tucker et al., 2010; Wittmann et al., 2014b). In addition, it has been shown that ectopic overexpression of Sox2 in the lateral olfactory epithelium enhances neurogenesis and significantly increases the generation of post-mitotic neurons (Tucker et al., 2010). Notably, at adult stages, Sox2 expression is maintained in stem cells in the olfactory epithelium (Amador-Arjona et al., 2015; Favaro et al., 2009; Guo et al., 2010; Kang and Hébert, 2012; Komitova and Eriksson, 2004; Packard et al., 2016), and the olfactory epithelium has the potential to recover almost completely after injury (reviewed by Schwob, 2002). A recent publication using an olfactory epithelial regenerative assay in adult *Sox2* conditional knockout mice, has shown that deletion of *Sox2* reduces the production of olfactory sensory neurons during regeneration and suggests that Sox2 expands the pool of neuronal progenitors (Packard et al., 2016). These data are consistent with our findings at embryonic stages that Sox2 is required for neurogenesis in the olfactory epithelium to establish and/or maintain neuronal progenitors during normal development. Taken together, these data indicate that Sox2 is essential for the neurogenic capacity to establish, maintain and expand the neuronal progenitor pool.

Another study has suggested that both SoxB1 members and Notch signalling play key roles during the maintenance of neural precursors in the CNS (Holmberg et al., 2008). Moreover, in the otic placodes, early Notch activity is required to maintain and restrict Sox2 expression to establish the pro-sensory otic region (Dabdoub et al., 2008; Neves et al., 2011). We now show that *Sox2* is expressed in the olfactory placode prior to *Notch1* and *Delta1* (*Dll1*) expression. Ours and other studies have shown that at later stages, from E10.5 and onwards, *Notch1* and *Delta1* expression are increased in the neurogenic part of the nasal epithelium (Cau et al., 2002; Schwarting et al., 2007; Wittmann et al., 2014b), which indicate that Notch activity is required to maintain and/or restrict ongoing olfactory neurogenesis. In addition, our results show that *Hes5* is already expressed in prospective olfactory epithelial cells of the head ectoderm at E9.0, prior to the expression of other neuronal markers such as *Ascl1*, *Ngn1*, *Neurod1* and Tuj1, which is in agreement with the expression pattern in chick (Maier and Gunhaga, 2009). Thus, *Hes5* appears to be the earliest known marker associated with neuronal determination of the olfactory epithelium. Previous studies in mice, at around E10.5-E12.5, have presented a model of crossregulation between *Hes1*, *Hes5* and *Ascl1* for the control of neuronal differentiation in the olfactory epithelium (Cau et al., 2002, 2000, 1997). *Hes1* has been suggested to regulate the neural progenitor domain, including *Ascl1* transcription in the olfactory placode, whereas *Hes5* expression is suggested to be dependent on *Ascl1* activity, likely via the Notch pathway (Cau et al., 2000). Our results, presenting the expression of *Hes5* at E9.0 in the absence of *Ascl1*, indicate that the initial upregulation of *Hes5* is independent of *Ascl1* activity. In addition, although *Hes5* expression is reduced in *Ascl1* knockout mice, there are domains of *Hes5*-positive cells and ongoing neurogenesis present in the *Ascl1*-deficient olfactory epithelium (Cau et al., 2000, 1997). It is possible that the remaining *Hes5*-positive cells and neurogenic olfactory domain in the *Ascl1* knockout mice are generated from the early *Ascl1*-independent *Hes5* cells, and that, at later stages, the regulation of *Hes5* expression and ongoing olfactory neurogenesis involves an *Ascl1* and Notch pathway control mechanism. The fact that all three individual knockout mice of *Hes1*, *Hes5* and *Ascl1* generate olfactory neurons (Cau et al., 2002, 2000, 1997) points to redundant functions of these genes during olfactory neurogenesis and that other upstream molecules are important to determine the olfactory neurogenic domain.

Our study suggests that *Sox2* is activating *Hes5* expression by regulating the Hes5 promoter. We show that disruption of Sox2 activity in the olfactory epithelium, part of the PNS, and the retina, part of the CNS, results in loss of *Hes5* expression. In agreement with this, *Hes5* expression is suppressed in conditionally ablated Sox2 retinal cells in mouse (Taranova et al., 2006). Interestingly, regulation of the *Hes5* promoter by SoxC transcription factors (Sox4, Sox11, Sox12) was recently proposed to mediate a crucial role for retinogenesis (Kuwajima et al., 2017). As Sox2 has been shown to bind neural lineage-specific genes, which later are bound and activated by SoxC factors in differentiating neurons (Bergsland et al., 2011), our observation might point to *Hes5* as a possible mediator of Sox2 function also in retinogenesis. Our mining of six available ChIP-Seq data sets (Hagey et al., 2016; Kwan et al., 2015; Lodato et al., 2013; Ng et al., 2013; Sancho-Martinez et al., 2016) revealed a peak of Sox2 binding to the *Hes5* promoter in neural progenitors in both the CNS and PNS in human and mouse cells. In agreement with our findings, a study in zebrafish using chromatin immunoprecipitation analysis of the Hes-class bHLH gene *her3* and *hesx1* genes suggests a direct regulation of these genes by SoxB1 members (Okuda et al., 2010). In the same study, a quadruple knockdown of the four SoxB1 genes *sox2*/*sox3*/*sox19a*/*sox19b* followed by gene expression analyses indicated that the SoxB1 members promote neural differentiation by regulating the Hes-class bHLH gene *her3* and the proneural-class bHLH genes *ngn1* (Okuda et al., 2010). In addition, a study of the inner ear in chick has suggested that Sox2 (a SoxB1 family member) activates Ngn1, which in turn act as a repressor for Hes genes (Evsen et al., 2013). This mechanism might also regulate neurogenesis in the olfactory epithelium. Although in the *Sox2* cKO olfactory placode, *Hes5* and *Ngn1* expression was never detected, indicating that, in the absence of *Sox2*, cells of the neuronal lineage are not specified in the olfactory epithelium. In a similar fashion, it has been suggested that *Sox2* directly activates the transcription of the bHLH proneural gene *Atoh1* (previously *Math1*) (Bermingham et al., 1999; Woods et al., 2004) to define the sensory domain of the otic epithelium (Neves et al., 2012). In conclusion, our findings indicate that the establishment of sensory progenitor cells in the olfactory epithelium requires Sox2-directed downregulation of *Bmp4* and upregulation of *Hes5* expression.

## MATERIALS AND METHODS

### Mouse and chick embryos

The conditional *Sox2-*deficient mouse line (*Sox2* cKO), described previously (Ferri et al., 2013), was generated by crossing *Sox2^flox/+^* mice (Favaro et al., 2009) with *Foxg1-Cre* mice (Hébert and McConnell, 2000). *Foxg1*-mediated *loxP* recombination in *Sox2* mutants occurred in the telencephalon and discrete head regions, including the olfactory epithelium. The generation, genotyping and phenotyping of *Sox2* cKO mice and their control littermates were performed as previously described (Ferri et al., 2013). The use of *Sox2* cKO and control mice was part of experimental protocols approved by the Italian Ministry of Health according to Legislative Decree 116, conforming to European legislation (authorization number 357/2016-PR). Fertilized white Lohman chicken eggs were obtained from Strömbäcks Ägg (Vännäs, Sweden). Chick embryos were staged according to the protocol of Hamburger and Hamilton (1951).

### *In situ* hybridization and immunohistochemistry

For the use of *in situ* RNA hybridization and immunohistochemistry, embryos were fixed in 4% PFA, transferred to 25% sucrose, embedded and stored at −80°C until cryosectioned at 10 µm on consecutive slides. *In situ* RNA hybridization was performed essentially as previously described (Wilkinson and Nieto, 1993). Applied mouse digoxigenin-labelled probes were: *Sox2* (Ferri et al., 2004), *Hes5* (Machold et al., 2007), *Ngn1* (a gift from G. Fishell, New York University, USA), *Neurod1* (Cau et al., 1997), *Msx2* (Iulianella et al., 2003), *Bmp4* (Iulianella et al., 2003) and *Fgf8* (a gift from H. Edlund, Umeå University, Sweden). Applied chick digoxigenin-labelled probes were: *Bmp4* (Francis et al., 1994)*, Dlx3*, *Dlx5* and *Hes5* (*Hes5-1*) (Fior and Henrique, 2005), *Ngn1* (Perez et al., 1999) and *NeuroD* (Bell et al., 2008).

Immunohistochemistry was performed using standard protocols. Antibodies used were: anti-Tuj1 (neuronal class III β-Tubulin, 1:500, Covance, MMS-435P), anti-Msx1/2 (1:10, DSHB, 4G1), anti-Ki67 (1:1000, Becton Dickinson, 556003), anti-BrdU (1:50, DSHB, G3G4), anti-Sox2 (1:1000, a gift from T. Edlund, Umeå University, Sweden), anti-GFP (1:600, Aves Labs, 1010), anti-cleaved caspase 3 (1:1000, Cell Signaling, 9961) and anti-phospho-Histone H3 (1:500, Millipore, 06-570). Alexa Fluor secondary antibodies (1:400, Molecular Probes, A32723, A11034, A11032) were used and nuclei were stained using DAPI (1:400-600, Sigma, D-9542). Briefly, sections were blocked in 10% foetal calf serum (FCS) prior to primary antibody incubation overnight at 4°C.

The protocol for BrdU/EdU histochemistry was as follows. Prior to blocking and antibody incubation, sections used for BrdU/EdU histochemistry were washed briefly in TBST at room temperature, for 10 min in ice-cold 1 M HCl at 4°C, for 20 min in 2 M HCl at 37°C, for 10 min in 0.1 M sodium borate buffer (pH 8.5) at room temperature and briefly in TBST at room temperature. Blocking was performed in 10% FCS followed by incubation with anti-BrdU antibody overnight at room temperature. EdU detection was performed according to the manufacturer's protocol (Molecular Probes). *In situ* and immunohistochemistry slides were mounted with glycerol or fluorescent mounting medium (Dako), respectively.

### Determination of cell cycle parameters in mouse embryos

E9.5 pregnant dams were first injected with bromodeoxyuridine (BrdU, Sigma) and 1.5 hours (h) later injected with ethynyldeoxyuridine (EdU, Molecular Probes) for 30 min, both at 50 µg/g body weight, before embryo collection. BrdU/EdU histochemistry details are given above. The cell cycle parameters were determined as previously described (Martynoga et al., 2005; Quinn et al., 2007). The 2 h BrdU pulse labelled nuclei in S phase and G2/M phases, whereas the 30 min EdU pulse labelled S-phase nuclei. The BrdU^+^/EdU^−^ nuclei correspond to cells leaving S phase during the 1.5 h period. The S phase (Ts) was calculated using the formula: Ts=1.5×(number of EdU^+^ cells/number of BrdU^+^/EdU^−^ cells). The total cell cycle length (Tc) was calculated as: Tc=Ts×(total cell number/number of EdU^+^ cells) (Martynoga et al., 2005; Quinn et al., 2007). At E9.5, Ki67 immunostaining, which defines proliferating cells (Yu et al., 1992), indicated that virtually all olfactory placodal cells proliferate (Fig. S10). Subsequently, the total number of proliferating cells was estimated by counting all DAPI nuclei in the placode area.

### CRISPR/Cas9 targeting of Sox2

The construct pUC19-U6-*Sox2*-gRNA was engineered to overexpress a CRISPR guide (g)RNA directed to the Sox2 locus under the control of the U6 promoter. Briefly, the gRNA 5′-GTTTTAGAGCTAGAAATAGCAAG TTAAAATAAGGCTAGTCCGTTATCAACTTGAAAAAGTGGCACCGAGTCGGTGCTTTTTTT-3′ (Garneau et al., 2010) was cloned in a pUC19 vector (Invitrogen). A 20 bp target sequence, corresponding to positions 537-556 of the chicken *Sox2*-coding sequence (sequence below) was cloned in front of the gRNA in the pUC19 vector by *Bbs*I digestion. Oligonucleotides for the *Sox2*-CRISPR construct were as follows: *Sox2*-forward, 5′-GGGGGCGGGAGGTTTCAGCT-3′; *Sox2*-reverse: 5′-AGCTGAAACCTCCCGCCCCC-3′.

The control construct pUC19-U6-*Cont*-gRNA was designed in a similar way replacing the 20 bp target sequence by a random nucleotide sequence as follows: *Cont*-gRNA-forward, 5′-GGACTGCTACGATCTACACC-3′; *Cont*-gRNA-reverse, 5′-GGTGTAGATCGTAGCAGTCC-3′

The oligonucleotides were diluted to a final 2 mM concentration in annealing buffer containing 100 mM potassium acetate and 30 mM Hepes (pH 7.4), denatured at 95°C for 3 min and cooled to 37°C for annealing over 2-3 h. The sequence of the construct was verified by Sanger sequencing.

### *In ovo* electroporation of chick embryos

Stage 8-10 chick embryos were electroporated in the olfactory placode region, and stage 10/11 chick embryos were electroporated in the head ectoderm in and around the olfactory placodal region, or the prospective retina by applying three pulses (9-12 V, 25 ms duration, 1 s interval), adapted to previous experiences (Wittmann et al., 2014a). Vectors used were: pCAβ-*EGFP*m5 (Yaneza et al., 2002), pCAG-hCas9 vector (addgene # 51142), pUC19-*Sox2*gRNA, pUC19-*Cont*-gRNA, pCAGGS-*Hes5* (Holmberg et al., 2008) and pMiwIII–Noggin (Timmer et al., 2002), all at a concentration of 1.0 μg/μl (Timmer et al., 2002). Inhibition of BMP signalling by the Noggin construct has previously been verified (Maier et al., 2010; Pandit et al., 2011). The constructs were transferred using an Electro Square Porator ECM 830 (BTX). After electroporation, the eggs were re-incubated to approximately stage 20-22 (olfactory epithelium) or stage 24 (retina). Viable embryos with GFP expression in the region of interest were selected for further analysis.

### Promoter analysis and ChIP-seq data mining

The 5-prime upstream sequence of mouse, human, chick and ostrich were retrieved from the current assembly available at Ensembl and Avianbase. Putative Sox2-binding sites were predicted using the PROMO tool (Farre et al., 2003). The alignment in Fig. S4 was performed using MAFFT (Katoh and Standley, 2013). The predicted Sox2-binding sequence was compared with consensus Sox2-binding sequences derived from the JASPAR database (Mathelier et al., 2016).

For analysis of available ChIP-seq data, pre-computed coverage tracks were obtained from the Cistrome database (cistrome.org/db/) and viewed in the UCSC genome browser. The datasets with SRA accession numbers SRR945967, SRR1929985, SRR1616842, SRR3151474, SRR3151475 and SRR630003 were used. For data not available on cistrome.org, raw reads were mapped and processed into coverage track files as specified on the Cistrome homepage (cistrome.org/db/#/about).

### Luciferase constructs

A wild-type 278 bp region, including four Sox2-binding sites, of the mouse *Hes5* promoter and a mutated *Hes5* promoter were cloned immediately upstream of the luciferase gene into the TK-LUC vector, from which the minimal TK promoter had been deleted (Ferri et al., 2013). In the mutated *Hes5* promoter, all four Sox2-binding sites (underlined) were mutated by changing 3 bp/binding site (italic): 5′-GGCGCGGGGCTCTCAGCATCAGGCCCCGGGATGCTAATGAGGGCGAGCGCGTTCCCACAGCCCGGACA*CCC*TGCCGCGCGGCCCACCTGCTCCTCGGGGAGCGACCA*CCC*TGCCCGCGCCAATTCACAGGCAATTTAGCGTGCGCTAATGGGCCGGCGCCT*CCC*TGCGGCCGGCGCCGCCA*CCC*GCCGCCGAGTGTGGGAACGGCCGCGGCGCCCGGACCCCAGGCGCCGGGCCGCTGCCCGCGCCTATATAGGGCTGGCGTGCTGGGGTCCAGGTCG 3′ (ordered from Sigma).

### Transfection experiments

Transfection experiments were performed essentially as previously described (Mariani et al., 2012). Specifically, Neuro-2a (N2a) cells were plated in Minimum Essential Medium Eagle (MEM; Sigma), supplemented with 10% foetal bovine serum, L-glutamine, penicillin and streptomycin. For transfection, cells were plated in 12-well-plates at 1.5×10^5^ cells/well, and transfected the following day with Lipofectamine 2000 (Invitrogen). According to the manufacturer's instructions medium in each well was replaced with 1 ml of MEM medium (with no addition) mixed with 2 µl of Lipofectamine 2000, and DNA. We used a fixed amount of 300 ng of luciferase reporter plasmid for each well, with increasing amount of *Sox2*-expressing vector, or *Mash1*-expressing vector (Favaro et al., 2009; Mariani et al., 2012), or the corresponding control ‘empty’ vectors (not containing the transcription factor's cDNA), in the following luciferase vector: expressing vector molar ratios (indicated as in Fig. 6 and Fig. S7): |, 1:0.025; ⊢, 1: 0.050; +, 1:0.075; ++, 1:0.125; +++, 1:0.25; ++++, 1:0.5. The pBluescript vector was added to each transfection to equalize the total amount of transfected DNA to a total of 800 ng in each reaction. After 24 h, total cellular extracts were prepared and Luciferase activity was measured with a Promega Luciferase Assay System, according to the manufacturer's instructions.

### Statistical analysis and imaging

The total number of Tuj1^+^ and aCaspase3^+^ cells was determined by counting the number of DAPI^+^ nuclei. The graphs represent the mean number±s.e.m. as percentage of the total cell number if not stated otherwise. Significance was determined using Student's *t*-test with *P<0.05, **P<0.01 and ***P<0.0001 accepted as statistically significant. Quantification and image generation was performed using a Nikon Eclipse E800 microscope for simultaneous Epi-fluorescence/DIC observations, equipped with a CCD camera connected to a PC (Nikon Imaging Software NIS-Elements). Images were processed using Photoshop CS2 (Adobe). BrdU^+^ and BrdU/EdU double-labelled cells were counted in the E9.5 olfactory placode. *Sox2* cKO mutant embryos were compared with age-matched wild-type and heterozygous *Sox2^flox/+^* littermates. All data were analysed using Prism GraphPad software. For the transfection assay, results are presented as the mean±s.e.m.

## Supplementary Material

Supplementary information

## References

[DEV153791C1] Amador-Arjona A., Cimadamore F., Huang C.-T., Wright R., Lewis S., Gage F. H. and Terskikh A. V. (2015). SOX2 primes the epigenetic landscape in neural precursors enabling proper gene activation during hippocampal neurogenesis. *Proc. Natl. Acad. Sci. USA* 112, E1936-E1945. 10.1073/pnas.1421480112 25825708 PMC4403144

[DEV153791C2] Avilion A. A., Nicolis S. K., Pevny L. H., Perez L., Vivian N. and Lovell-Badge R. (2003). Multipotent cell lineages in early mouse development depend on SOX2 function. *Genes Dev.* 17, 126-140. 10.1101/gad.224503 12514105 PMC195970

[DEV153791C3] Bell D., Streit A., Gorospe I., Varela-Nieto I., Alsina B. and Giraldez F. (2008). Spatial and temporal segregation of auditory and vestibular neurons in the otic placode. *Dev. Biol.* 322, 109-120. 10.1016/j.ydbio.2008.07.011 18674529

[DEV153791C4] Bergsland M., Ramskold D., Zaouter C., Klum S., Sandberg R. and Muhr J. (2011). Sequentially acting Sox transcription factors in neural lineage development. *Genes Dev.* 25, 2453-2464. 10.1101/gad.176008.111 22085726 PMC3243056

[DEV153791C5] Bermingham N. A., Hassan B. A., Price S. D., Vollrath M. A., Ben-Arie N., Eatock R. A., Bellen H. J., Lysakowski A. and Zoghbi H. Y. (1999). Math1: an essential gene for the generation of inner ear hair cells. *Science* 284, 1837-1841. 10.1126/science.284.5421.1837 10364557

[DEV153791C6] Bertrand N., Castro D. S. and Guillemot F. (2002). Proneural genes and the specification of neural cell types. *Nat. Rev. Neurosci.* 3, 517-530. 10.1038/nrn874 12094208

[DEV153791C7] Bhattacharyya S. and Bronner-Fraser M. (2008). Competence, specification and commitment to an olfactory placode fate. *Development* 135, 4165-4177. 10.1242/dev.026633 19029046

[DEV153791C8] Bonaguidi M. A., Peng C.-Y., McGuire T., Falciglia G., Gobeske K. T., Czeisler C. and Kessler J. A. (2008). Noggin expands neural stem cells in the adult hippocampus. *J. Neurosci.* 28, 9194-9204. 10.1523/JNEUROSCI.3314-07.2008 18784300 PMC3651371

[DEV153791C9] Brann J. H. and Firestein S. J. (2014). A lifetime of neurogenesis in the olfactory system. *Front. Neurosci.* 8, 182 10.3389/fnins.2014.00182 25018692 PMC4071289

[DEV153791C10] Bylund M., Andersson E., Novitch B. G. and Muhr J. (2003). Vertebrate neurogenesis is counteracted by Sox1-3 activity. *Nat. Neurosci.* 6, 1162-1168. 10.1038/nn1131 14517545

[DEV153791C11] Cau E., Gradwohl G., Fode C. and Guillemot F. (1997). Mash1 activates a cascade of bHLH regulators in olfactory neuron progenitors. *Development* 124, 1611-1621. 9108377 10.1242/dev.124.8.1611

[DEV153791C12] Cau E., Gradwohl G., Casarosa S., Kageyama R. and Guillemot F. (2000). Hes genes regulate sequential stages of neurogenesis in the olfactory epithelium. *Development* 127, 2323-2332. 10804175 10.1242/dev.127.11.2323

[DEV153791C13] Cau E., Casarosa S. and Guillemot F. (2002). Mash1 and Ngn1 control distinct steps of determination and differentiation in the olfactory sensory neuron lineage. *Development* 129, 1871-1880. 11934853 10.1242/dev.129.8.1871

[DEV153791C14] Cavallaro M., Mariani J., Lancini C., Latorre E., Caccia R., Gullo F., Valotta M., DeBiasi S., Spinardi L., Ronchi A. et al. (2008). Impaired generation of mature neurons by neural stem cells from hypomorphic Sox2 mutants. *Development* 135, 541-557. 10.1242/dev.010801 18171687

[DEV153791C15] Chen S., Li X., Lu D., Xu Y., Mou W., Wang L., Chen Y., Liu Y., Li X., Li L.-Y. et al. (2014). SOX2 regulates apoptosis through MAP4K4-survivin signaling pathway in human lung cancer cells. *Carcinogenesis* 35, 613-623. 10.1093/carcin/bgt371 24233838

[DEV153791C16] Croucher S. J. and Tickle C. (1989). Characterization of epithelial domains in the nasal passages of chick embryos: spatial and temporal mapping of a range of extracellular matrix and cell surface molecules during development of the nasal placode. *Development* 106, 493-509. 2480879 10.1242/dev.106.3.493

[DEV153791C17] Dabdoub A., Puligilla C., Jones J. M., Fritzsch B., Cheah K. S. E., Pevny L. H. and Kelley M. W. (2008). Sox2 signaling in prosensory domain specification and subsequent hair cell differentiation in the developing cochlea. *Proc. Natl. Acad. Sci. USA* 105, 18396-18401. 10.1073/pnas.0808175105 19011097 PMC2587543

[DEV153791C18] Ellis P., Fagan B. M., Magness S. T., Hutton S., Taranova O., Hayashi S., McMahon A., Rao M. and Pevny L. (2004). SOX2, a persistent marker for multipotential neural stem cells derived from embryonic stem cells, the embryo or the adult. *Dev. Neurosci.* 26, 148-165. 10.1159/000082134 15711057

[DEV153791C19] Evsen L., Sugahara S., Uchikawa M., Kondoh H. and Wu D. K. (2013). Progression of neurogenesis in the inner ear requires inhibition of Sox2 transcription by neurogenin1 and neurod1. *J. Neurosci.* 33, 3879-3890. 10.1523/JNEUROSCI.4030-12.2013 23447599 PMC3865497

[DEV153791C20] Farre D., Roset R., Huerta M., Adsuara J. E., Rosello L., Alba M. M. and Messeguer X. (2003). Identification of patterns in biological sequences at the ALGGEN server: PROMO and MALGEN. *Nucleic Acids Res.* 31, 3651-3653. 10.1093/nar/gkg605 12824386 PMC169011

[DEV153791C21] Favaro R., Valotta M., Ferri A. L. M., Latorre E., Mariani J., Giachino C., Lancini C., Tosetti V., Ottolenghi S., Taylor V. et al. (2009). Hippocampal development and neural stem cell maintenance require Sox2-dependent regulation of Shh. *Nat. Neurosci.* 12, 1248-1256. 10.1038/nn.2397 19734891

[DEV153791C22] Feng R. and Wen J. (2015). Overview of the roles of Sox2 in stem cell and development. *Biol. Chem.* 396, 883-891. 10.1515/hsz-2014-0317 25781683

[DEV153791C23] Feng R., Zhou S., Liu Y., Song D., Luan Z., Dai X., Li Y., Tang N., Wen J. and Li L. (2013). Sox2 protects neural stem cells from apoptosis via up-regulating survivin expression. *Biochem. J.* 450, 459-468. 10.1042/BJ20120924 23301561

[DEV153791C24] Ferri A. L., Cavallaro M., Braida D., Di Cristofano A., Canta A., Vezzani A., Ottolenghi S., Pandolfi P. P., Sala M., DeBiasi S. et al. (2004). Sox2 deficiency causes neurodegeneration and impaired neurogenesis in the adult mouse brain. *Development* 131, 3805-3819. 10.1242/dev.01204 15240551

[DEV153791C25] Ferri A., Favaro R., Beccari L., Bertolini J., Mercurio S., Nieto-Lopez F., Verzeroli C., La Regina F., De Pietri Tonelli D., Ottolenghi S. et al. (2013). Sox2 is required for embryonic development of the ventral telencephalon through the activation of the ventral determinants Nkx2.1 and Shh. *Development* 140, 1250-1261. 10.1242/dev.073411 23444355

[DEV153791C26] Fior R. and Henrique D. (2005). A novel hes5/hes6 circuitry of negative regulation controls Notch activity during neurogenesis. *Dev. Biol.* 281, 318-333. 10.1016/j.ydbio.2005.03.017 15893982

[DEV153791C27] Fletcher R. B., Prasol M. S., Estrada J., Baudhuin A., Vranizan K., Choi Y. G. and Ngai J. (2011). p63 regulates olfactory stem cell self-renewal and differentiation. *Neuron* 72, 748-759. 10.1016/j.neuron.2011.09.009 22153372 PMC3240811

[DEV153791C28] Fornaro M., Geuna S., Fasolo A. and Giacobini-Robecchi M. G. (2001). Evidence of very early neuronal migration from the olfactory placode of the chick embryo. *Neuroscience* 107, 191-197. 10.1016/S0306-4522(01)00334-7 11731093

[DEV153791C29] Fornaro M., Geuna S., Fasolo A. and Giacobini-Robecchi M. G. (2003). HuC/D confocal imaging points to olfactory migratory cells as the first cell population that expresses a post-mitotic neuronal phenotype in the chick embryo. *Neuroscience* 122, 123-128. 10.1016/j.neuroscience.2003.07.004 14596854

[DEV153791C30] Francis P. H., Richardson M. K., Brickell P. M. and Tickle C. (1994). Bone morphogenetic proteins and a signalling pathway that controls patterning in the developing chick limb. *Development* 120, 209-218. 8119128 10.1242/dev.120.1.209

[DEV153791C31] Garneau J. E., Dupuis M.-E., Villion M., Romero D. A., Barrangou R., Boyaval P., Fremaux C., Horvath P., Magadán A. H. and Moineau S. (2010). The CRISPR/Cas bacterial immune system cleaves bacteriophage and plasmid DNA. *Nature* 468, 67-71. 10.1038/nature09523 21048762

[DEV153791C32] Graham V., Khudyakov J., Ellis P. and Pevny L. (2003). SOX2 functions to maintain neural progenitor identity. *Neuron* 39, 749-765. 10.1016/S0896-6273(03)00497-5 12948443

[DEV153791C33] Guo Z., Packard A., Krolewski R. C., Harris M. T., Manglapus G. L. and Schwob J. E. (2010). Expression of pax6 and sox2 in adult olfactory epithelium. *J. Comp. Neurol.* 518, 4395-4418. 10.1002/cne.22463 20852734 PMC2940252

[DEV153791C34] Hagey D. W. and Muhr J. (2014). Sox2 acts in a dose-dependent fashion to regulate proliferation of cortical progenitors. *Cell Rep.* 9, 1908-1920. 10.1016/j.celrep.2014.11.013 25482558

[DEV153791C35] Hagey D. W., Zaouter C., Combeau G., Lendahl M. A., Andersson O., Huss M. and Muhr J. (2016). Distinct transcription factor complexes act on a permissive chromatin landscape to establish regionalized gene expression in CNS stem cells. *Genome Res.* 26, 908-917. 10.1101/gr.203513.115 27197220 PMC4937566

[DEV153791C36] Hamburger V. and Hamilton H. L. (1951). A series of normal stages in the development of the chick embryo. *J. Morphol.* 88, 49-92. 10.1002/jmor.1050880104 24539719

[DEV153791C37] Hébert J. M. and McConnell S. K. (2000). Targeting of cre to the Foxg1 (BF-1) locus mediates loxP recombination in the telencephalon and other developing head structures. *Dev. Biol.* 222, 296-306. 10.1006/dbio.2000.9732 10837119

[DEV153791C38] Hille F. and Charpentier E. (2016). CRISPR-Cas: biology, mechanisms and relevance. *Philos. Trans. R. Soc. Lond. B Biol. Sci.* 371, 1707 10.1098/rstb.2015.0496 PMC5052741 27672148

[DEV153791C39] Holmberg J., Hansson E., Malewicz M., Sandberg M., Perlmann T., Lendahl U. and Muhr J. (2008). SoxB1 transcription factors and Notch signaling use distinct mechanisms to regulate proneural gene function and neural progenitor differentiation. *Development* 135, 1843-1851. 10.1242/dev.020180 18417619

[DEV153791C40] Iso T., Kedes L. and Hamamori Y. (2003). HES and HERP families: multiple effectors of the Notch signaling pathway. *J. Cell. Physiol.* 194, 237-255. 10.1002/jcp.10208 12548545

[DEV153791C41] Iulianella A., Vanden Heuvel G. and Trainor P. (2003). Dynamic expression of murine Cux2 in craniofacial, limb, urogenital and neuronal primordia. *Gene Expr. Patterns* 3, 571-577. 10.1016/S1567-133X(03)00123-6 12971989

[DEV153791C42] Jidigam V. K., Srinivasan R. C., Patthey C. and Gunhaga L. (2015). Apical constriction and epithelial invagination are regulated by BMP activity. *Biol. Open* 4, 1782-1791. 10.1242/bio.015263 26621830 PMC4736041

[DEV153791C43] Kageyama R. and Ohtsuka T. (1999). The Notch-Hes pathway in mammalian neural development. *Cell Res.* 9, 179-188. 10.1038/sj.cr.7290016 10520600

[DEV153791C44] Kam J. W. K., Raja R. and Cloutier J.-F. (2014). Cellular and molecular mechanisms regulating embryonic neurogenesis in the rodent olfactory epithelium. *Int. J. Dev. Neurosci.* 37, 76-86. 10.1016/j.ijdevneu.2014.06.017 25003986

[DEV153791C45] Kam J. W. K., Dumontier E., Baim C., Brignall A. C., Mendes da Silva D., Cowan M., Kennedy T. E. and Cloutier J.-F. (2016). RGMB and neogenin control cell differentiation in the developing olfactory epithelium. *Development* 143, 1534-1546. 10.1242/dev.118638 27143755

[DEV153791C46] Kang W. and Hébert J. M. (2012). A Sox2 BAC transgenic approach for targeting adult neural stem cells. *PLoS ONE* 7, e49038 10.1371/journal.pone.0049038 23145058 PMC3492187

[DEV153791C47] Katoh K. and Standley D. M. (2013). MAFFT multiple sequence alignment software version 7: improvements in performance and usability. *Mol. Biol. Evol.* 30, 772-780. 10.1093/molbev/mst010 23329690 PMC3603318

[DEV153791C48] Kawauchi S., Kim J., Santos R., Wu H.-H., Lander A. D. and Calof A. L. (2009). Foxg1 promotes olfactory neurogenesis by antagonizing Gdf11. *Development* 136, 1453-1464. 10.1242/dev.034967 19297409 PMC2674256

[DEV153791C49] Kazanis I. (2013). Neurogenesis in the adult mammalian brain: how much do we need, how much do we have? *Curr. Top. Behav. Neurosci.* 15, 3-29. 10.1007/7854_2012_227 22976273

[DEV153791C50] Kiernan A. E., Pelling A. L., Leung K. K. H., Tang A. S. P., Bell D. M., Tease C., Lovell-Badge R., Steel K. P. and Cheah K. S. E. (2005). Sox2 is required for sensory organ development in the mammalian inner ear. *Nature* 434, 1031-1035. 10.1038/nature03487 15846349

[DEV153791C51] Kohl Z., Regensburger M., Aigner R., Kandasamy M., Winner B., Aigner L. and Winkler J. (2010). Impaired adult olfactory bulb neurogenesis in the R6/2 mouse model of Huntington's disease. *BMC Neurosci.* 11, 114 10.1186/1471-2202-11-114 20836877 PMC2945356

[DEV153791C52] Komitova M. and Eriksson P. S. (2004). Sox-2 is expressed by neural progenitors and astroglia in the adult rat brain. *Neurosci. Lett.* 369, 24-27. 10.1016/j.neulet.2004.07.035 15380301

[DEV153791C53] Krolewski R. C., Packard A., Jang W., Wildner H. and Schwob J. E. (2012). Ascl1 (Mash1) knockout perturbs differentiation of nonneuronal cells in olfactory epithelium. *PLoS ONE* 7, e51737 10.1371/journal.pone.0051737 23284756 PMC3524087

[DEV153791C54] Kuwajima T., Soares C. A., Sitko A. A., Lefebvre V. and Mason C. (2017). SoxC transcription factors promote contralateral retinal ganglion cell differentiation and axon guidance in the mouse visual system. *Neuron* 93, 1110-1125.e5. 10.1016/j.neuron.2017.01.029 28215559 PMC5346053

[DEV153791C55] Kwan K. Y., Shen J. and Corey D. P. (2015). C-MYC transcriptionally amplifies SOX2 target genes to regulate self-renewal in multipotent otic progenitor cells. *Stem Cell Rep.* 4, 47-60. 10.1016/j.stemcr.2014.11.001 PMC4297878 25497456

[DEV153791C56] Lazic S. E., Grote H., Armstrong R. J. E., Blakemore C., Hannan A. J., van Dellen A. and Barker R. A. (2004). Decreased hippocampal cell proliferation in R6/1 Huntington's mice. *Neuroreport* 15, 811-813. 10.1097/00001756-200404090-00014 15073520

[DEV153791C57] Lodato M. A., Ng C. W., Wamstad J. A., Cheng A. W., Thai K. K., Fraenkel E., Jaenisch R. and Boyer L. A. (2013). SOX2 co-occupies distal enhancer elements with distinct POU factors in ESCs and NPCs to specify cell state. *PLoS Genet.* 9, e1003288 10.1371/journal.pgen.1003288 23437007 PMC3578749

[DEV153791C58] Machold R. P., Kittell D. and Fishell G. J. (2007). Antagonism between Notch and bone morphogenetic protein receptor signaling regulates neurogenesis in the cerebellar rhombic lip. *Neural Dev.* 2, 5 10.1186/1749-8104-2-5 17319963 PMC1820780

[DEV153791C59] Maier E. and Gunhaga L. (2009). Dynamic expression of neurogenic markers in the developing chick olfactory epithelium. *Dev. Dyn.* 238, 1617-1625. 10.1002/dvdy.21966 19441054

[DEV153791C60] Maier E., von Hofsten J., Nord H., Fernandes M., Paek H., Hebert J. M. and Gunhaga L. (2010). Opposing Fgf and Bmp activities regulate the specification of olfactory sensory and respiratory epithelial cell fates. *Development* 137, 1601-1611. 10.1242/dev.051219 20392740 PMC2860246

[DEV153791C61] Maier E., Nord H., von Hofsten J. and Gunhaga L. (2011). A balance of BMP and notch activity regulates neurogenesis and olfactory nerve formation. *PLoS ONE* 6, e17379 10.1371/journal.pone.0017379 21383851 PMC3044177

[DEV153791C62] Mariani J., Favaro R., Lancini C., Vaccari G., Ferri A. L., Bertolini J., Tonoli D., Latorre E., Caccia R., Ronchi A. et al. (2012). Emx2 is a dose-dependent negative regulator of Sox2 telencephalic enhancers. *Nucleic Acids Res.* 40, 6461-6476. 10.1093/nar/gks295 22495934 PMC3413107

[DEV153791C63] Martynoga B., Morrison H., Price D. J. and Mason J. O. (2005). Foxg1 is required for specification of ventral telencephalon and region-specific regulation of dorsal telencephalic precursor proliferation and apoptosis. *Dev. Biol.* 283, 113-127. 10.1016/j.ydbio.2005.04.005 15893304

[DEV153791C64] Masui S., Nakatake Y., Toyooka Y., Shimosato D., Yagi R., Takahashi K., Okochi H., Okuda A., Matoba R., Sharov A. A. et al. (2007). Pluripotency governed by Sox2 via regulation of Oct3/4 expression in mouse embryonic stem cells. *Nat. Cell Biol.* 9, 625-635. 10.1038/ncb1589 17515932

[DEV153791C65] Mathelier A., Fornes O., Arenillas D. J., Chen C.-Y., Denay G., Lee J., Shi W., Shyr C., Tan G., Worsley-Hunt R. et al. (2016). JASPAR 2016: a major expansion and update of the open-access database of transcription factor binding profiles. *Nucleic Acids Res.* 44, D110-D115. 10.1093/nar/gkv1176 26531826 PMC4702842

[DEV153791C66] Maucksch C., Jones K. S. and Connor B. (2013). Concise review: the involvement of SOX2 in direct reprogramming of induced neural stem/precursor cells. *Stem Cells Transl. Med.* 2, 579-583. 10.5966/sctm.2012-0179 23817132 PMC3726137

[DEV153791C67] Neves J., Parada C., Chamizo M. and Giraldez F. (2011). Jagged 1 regulates the restriction of Sox2 expression in the developing chicken inner ear: a mechanism for sensory organ specification. *Development* 138, 735-744. 10.1242/dev.060657 21266409

[DEV153791C68] Neves J., Uchikawa M., Bigas A. and Giraldez F. (2012). The prosensory function of Sox2 in the chicken inner ear relies on the direct regulation of Atoh1. *PLoS ONE* 7, e30871 10.1371/journal.pone.0030871 22292066 PMC3264626

[DEV153791C69] Ng S.-Y., Bogu G. K., Soh B. S. and Stanton L. W. (2013). The long noncoding RNA RMST interacts with SOX2 to regulate neurogenesis. *Mol. Cell* 51, 349-359. 10.1016/j.molcel.2013.07.017 23932716

[DEV153791C70] Ohtsuka T., Ishibashi M., Gradwohl G., Nakanishi S., Guillemot F. and Kageyama R. (1999). Hes1 and Hes5 as notch effectors in mammalian neuronal differentiation. *EMBO J.* 18, 2196-2207. 10.1093/emboj/18.8.2196 10205173 PMC1171303

[DEV153791C71] Okuda Y., Ogura E., Kondoh H. and Kamachi Y. (2010). B1 SOX coordinate cell specification with patterning and morphogenesis in the early zebrafish embryo. *PLoS Genet.* 6, e1000936 10.1371/journal.pgen.1000936 20463883 PMC2865518

[DEV153791C72] Packard A., Giel-Moloney M., Leiter A. and Schwob J. E. (2011). Progenitor cell capacity of NeuroD1-expressing globose basal cells in the mouse olfactory epithelium. *J. Comp. Neurol.* 519, 3580-3596. 10.1002/cne.22726 21800309 PMC4005605

[DEV153791C73] Packard A. I., Lin B. and Schwob J. E. (2016). Sox2 and Pax6 play counteracting roles in regulating neurogenesis within the murine olfactory epithelium. *PLoS ONE* 11, e0155167 10.1371/journal.pone.0155167 27171428 PMC4865097

[DEV153791C74] Pandit T., Jidigam V. K. and Gunhaga L. (2011). BMP-induced L-Maf regulates subsequent BMP-independent differentiation of primary lens fibre cells. *Dev. Dyn.* 240, 1917-1928. 10.1002/dvdy.22692 21761477

[DEV153791C75] Perez S. E., Rebelo S. and Anderson D. J. (1999). Early specification of sensory neuron fate revealed by expression and function of neurogenins in the chick embryo. *Development* 126, 1715-1728. 10079233 10.1242/dev.126.8.1715

[DEV153791C76] Pevny L. H. and Nicolis S. K. (2010). Sox2 roles in neural stem cells. *Int. J. Biochem. Cell Biol.* 42, 421-424. 10.1016/j.biocel.2009.08.018 19733254

[DEV153791C77] Pevny L. and Placzek M. (2005). SOX genes and neural progenitor identity. *Curr. Opin. Neurobiol.* 15, 7-13. 10.1016/j.conb.2005.01.016 15721738

[DEV153791C78] Quinn J. C., Molinek M., Martynoga B. S., Zaki P. A., Faedo A., Bulfone A., Hevner R. F., West J. D. and Price D. J. (2007). Pax6 controls cerebral cortical cell number by regulating exit from the cell cycle and specifies cortical cell identity by a cell autonomous mechanism. *Dev. Biol.* 302, 50-65. 10.1016/j.ydbio.2006.08.035 16979618 PMC2384163

[DEV153791C79] Ross S. E., Greenberg M. E. and Stiles C. D. (2003). Basic helix-loop-helix factors in cortical development. *Neuron* 39, 13-25. 10.1016/S0896-6273(03)00365-9 12848929

[DEV153791C80] Sancho-Martinez I., Nivet E., Xia Y., Hishida T., Aguirre A., Ocampo A., Ma L., Morey R., Krause M. N., Zembrzycki A. et al. (2016). Establishment of human iPSC-based models for the study and targeting of glioma initiating cells. *Nat. Commun.* 7, 10743 10.1038/ncomms10743 26899176 PMC4764898

[DEV153791C81] Sarlak G. and Vincent B. (2016). The roles of the stem cell-controlling Sox2 transcription factor: from neuroectoderm development to Alzheimer's disease? *Mol. Neurobiol.* 53, 1679-1698. 25691455 10.1007/s12035-015-9123-4

[DEV153791C82] Schwarting G. A., Gridley T. and Henion T. R. (2007). Notch1 expression and ligand interactions in progenitor cells of the mouse olfactory epithelium. *J. Mol. Histol.* 38, 543-553. 10.1007/s10735-007-9110-9 17605079

[DEV153791C83] Schwob J. E. (2002). Neural regeneration and the peripheral olfactory system. *Anat. Rec.* 269, 33-49. 10.1002/ar.10047 11891623

[DEV153791C84] Shimozaki K. (2014). Sox2 transcription network acts as a molecular switch to regulate properties of neural stem cells. *World J. Stem Cells* 6, 485-490. 10.4252/wjsc.v6.i4.485 25258670 PMC4172677

[DEV153791C85] Suh H., Consiglio A., Ray J., Sawai T., D'Amour K. A. and Gage F. H. (2007). In vivo fate analysis reveals the multipotent and self-renewal capacities of Sox2+ neural stem cells in the adult hippocampus. *Cell Stem Cell* 1, 515-528. 10.1016/j.stem.2007.09.002 18371391 PMC2185820

[DEV153791C86] Taranova O. V., Magness S. T., Fagan B. M., Wu Y., Surzenko N., Hutton S. R. and Pevny L. H. (2006). SOX2 is a dose-dependent regulator of retinal neural progenitor competence. *Genes Dev.* 20, 1187-1202. 10.1101/gad.1407906 16651659 PMC1472477

[DEV153791C87] Timmer J. R., Wang C. and Niswander L. (2002). BMP signaling patterns the dorsal and intermediate neural tube via regulation of homeobox and helix-loop-helix transcription factors. *Development* 129, 2459-2472. 11973277 10.1242/dev.129.10.2459

[DEV153791C88] Tripathi V.-B., Ishii Y., Abu-Elmagd M. M. and Scotting P. J. (2009). The surface ectoderm of the chick embryo exhibits dynamic variation in its response to neurogenic signals. *Int. J. Dev. Biol.* 53, 1023-1033. 10.1387/ijdb.082780vt 19598119

[DEV153791C89] Tucker E. S., Lehtinen M. K., Maynard T., Zirlinger M., Dulac C., Rawson N., Pevny L. and LaMantia A.-S. (2010). Proliferative and transcriptional identity of distinct classes of neural precursors in the mammalian olfactory epithelium. *Development* 137, 2471-2481. 10.1242/dev.049718 20573694 PMC2927697

[DEV153791C90] Urban N. and Guillemot F. (2014). Neurogenesis in the embryonic and adult brain: same regulators, different roles. *Front. Cell. Neurosci.* 8, 396 10.3389/fncel.2014.00396 25505873 PMC4245909

[DEV153791C91] Wei H., Lang M.-F. and Jiang X. (2013). Calretinin is expressed in the intermediate cells during olfactory receptor neuron development. *Neurosci. Lett.* 542, 42-46. 10.1016/j.neulet.2013.03.022 23537777

[DEV153791C92] Wilkinson D. G. and Nieto M. A. (1993). Detection of messenger RNA by in situ hybridization to tissue sections and whole mounts. *Methods Enzymol.* 225, 361-373. 10.1016/0076-6879(93)25025-W 8231863

[DEV153791C93] Wittmann W., Iulianella A. and Gunhaga L. (2014a). Cux2 acts as a critical regulator for neurogenesis in the olfactory epithelium of vertebrates. *Dev. Biol.* 388, 35-47. 10.1016/j.ydbio.2014.01.026 24512687

[DEV153791C94] Wittmann W., Schimmang T. and Gunhaga L. (2014b). Progressive effects of N-myc deficiency on proliferation, neurogenesis, and morphogenesis in the olfactory epithelium. *Dev. Neurobiol.* 74, 643-656. 10.1002/dneu.22162 24376126 PMC4237195

[DEV153791C95] Woods C., Montcouquiol M. and Kelley M. W. (2004). Math1 regulates development of the sensory epithelium in the mammalian cochlea. *Nat. Neurosci.* 7, 1310-1318. 10.1038/nn1349 15543141

[DEV153791C96] Xuan S., Baptista C. A., Balas G., Tao W., Soares V. C. and Lai E. (1995). Winged helix transcription factor BF-1 is essential for the development of the cerebral hemispheres. *Neuron* 14, 1141-1152. 10.1016/0896-6273(95)90262-7 7605629

[DEV153791C97] Yaneza M., Gilthorpe J. D., Lumsden A. and Tucker A. S. (2002). No evidence for ventrally migrating neural tube cells from the mid- and hindbrain. *Dev. Dyn.* 223, 163-167. 10.1002/dvdy.1241 11803580

[DEV153791C98] Yu C. C.-W., Woods A. L. and Levison D. A. (1992). The assessment of cellular proliferation by immunohistochemistry: a review of currently available methods and their applications. *Histochem. J.* 24, 121-131. 10.1007/BF01047461 1349881

[DEV153791C99] Zappone M. V., Galli R., Catena R., Meani N., De Biasi S., Mattei E., Tiveron C., Vescovi A. L., Lovell-Badge R., Ottolenghi S. et al. (2000). Sox2 regulatory sequences direct expression of a (beta)-geo transgene to telencephalic neural stem cells and precursors of the mouse embryo, revealing regionalization of gene expression in CNS stem cells. *Development* 127, 2367-2382. 10804179 10.1242/dev.127.11.2367

