# Supplementary material for: Sox2 is required for olfactory pit formation and olfactory neurogenesis through BMP restriction and Hes5 upregulation
Source: Development. 2018 Jan 15;145(2):dev153791. doi: 10.1242/dev.153791 (PMC5825848; doi:10.1242/dev.153791)
Supplement: Supplementary information [file develop-145-153791-s1.pdf]

Supplementary Figures

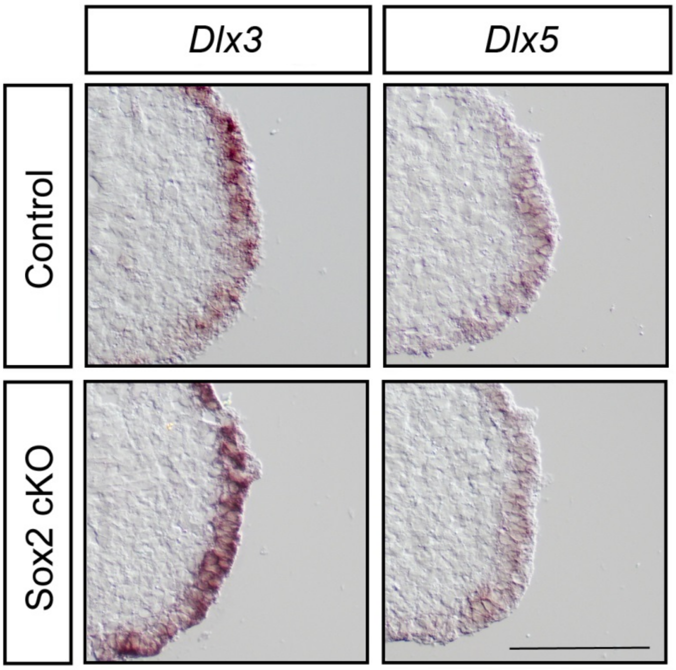

Fig. S1. Similar *Dlx3* and *Dlx5* expression patterns in the olfactory placode of wildtype and Sox2 cKO mice embryos.

At E9.5, no change in *Dlx3* or *Dlx5* expression can be detected in the olfactory placode of *Sox2* cKO embryos (n=3) compared to wildtype embryos (n=3). Scale bar: 100 µm.

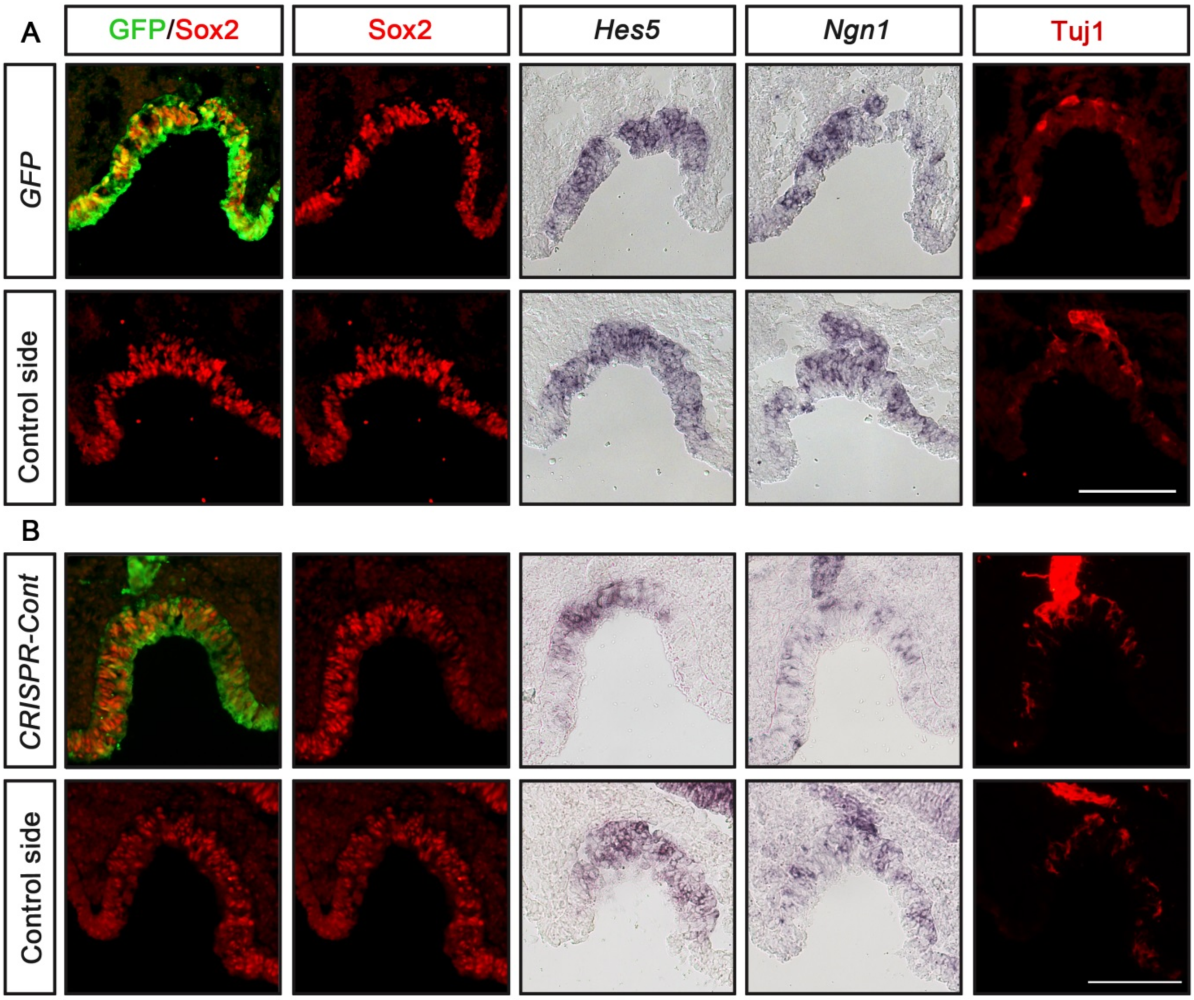

Fig. S2. Neither the *GFP* vector nor the CRISPR-*Cont* vector affects the development of the olfactory epithelium.

(A,B) In ovo electroporation of stage 9/10 chick embryos in the olfactory epithelium using a pCAβ-*EGFP*-m5 control vector alone (A; n=6) or together with pUC19-*Cont*-gRNA and pCAG-hCas9 vectors (B; n=5), and cultured to approximately stage 20-22 did not alter the morphology of the olfactory pit or the expression of Sox2, *Hes5*, *Ngn1* or Tuj1 compared to the non-electroporated control side. Scale bar: 100 µm.

Development • Supplementary information

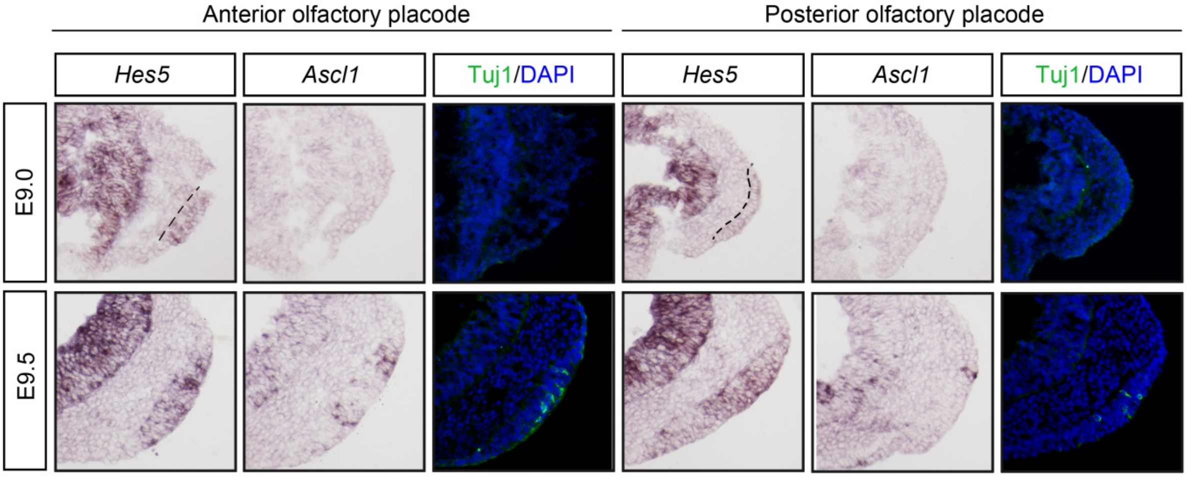

Fig. S3. *Hes5* expression precedes *Ascl1*expression in the mouse olfactory epithelium. At E9.0 in mouse, *Hes5,* but not *Ascl1* or Tuj1, is expressed in the olfactory placodal region (indicated by broken lines). By E9.5, *Hes5*, *Ascl1* and Tuj1 are expressed in the olfactory placode.

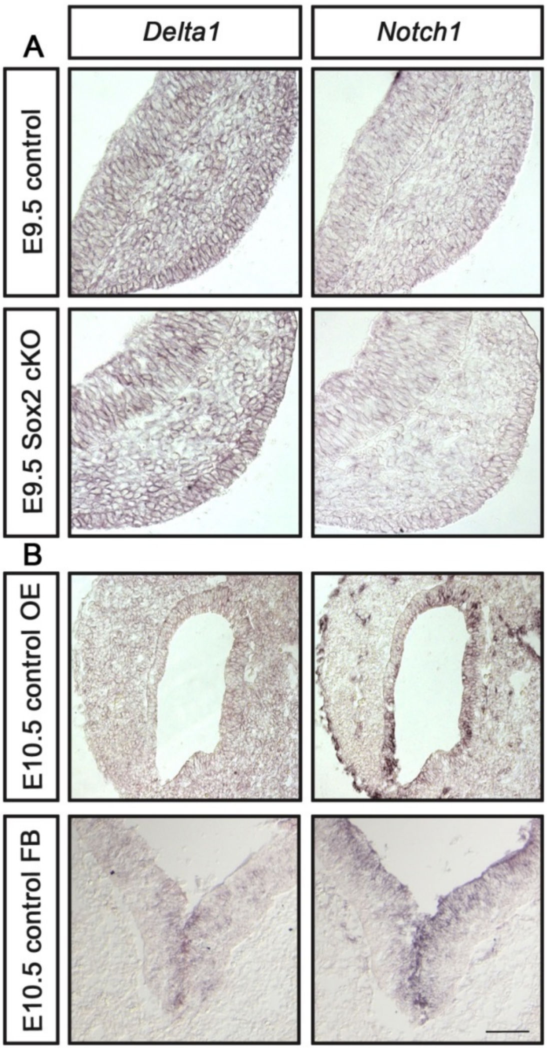

Fig. S4. Similar *Delta* and *Notch1* expression patterns in the olfactory epithelium of wildtype and Sox2 cKO mice embryos.

(A) No or weak expression of *Notch1* and *Delta1* are detected in the olfactory placode of wild-type and *Sox2* cKO embryos at E9.5. (B) *Notch1* expression and a few *Delta1*$^{+}$ cells are observed in the olfactory epithelium at E10.5 in wild-type embryos. *Notch1* and *Delta1* expression detected in the ventral forebrain at E10.5 in wild-type embryos are shown as positive controls for the probes. Scale bar: 100 µm.

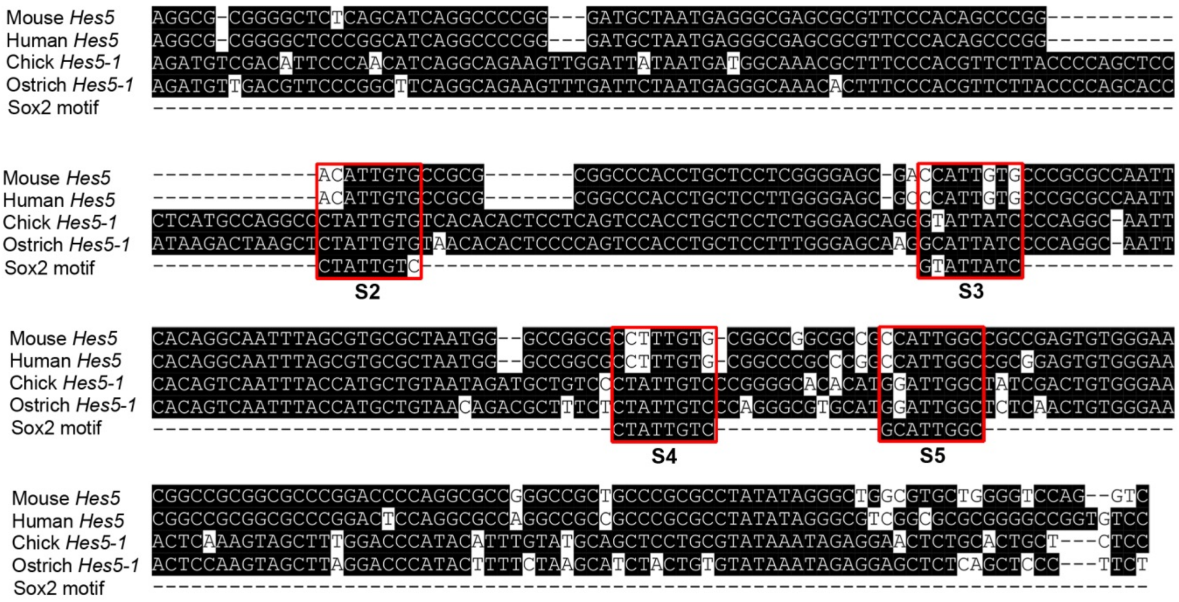

Fig. S5. Sequence alignment of the mouse, human, chick, and ostrich *Hes5* promoters.

The mouse *Hes5* sequence upstream of the transcription start site was aligned with the human, chick, and ostrich *Hes5* corresponding sequences. Four conserved putative Sox2-binding sites were identified with the transcription factor binding site prediction tool PROMO, indicated by red boxes. The consensus Sox2-binding motifs are also indicated.

Development • Supplementary information

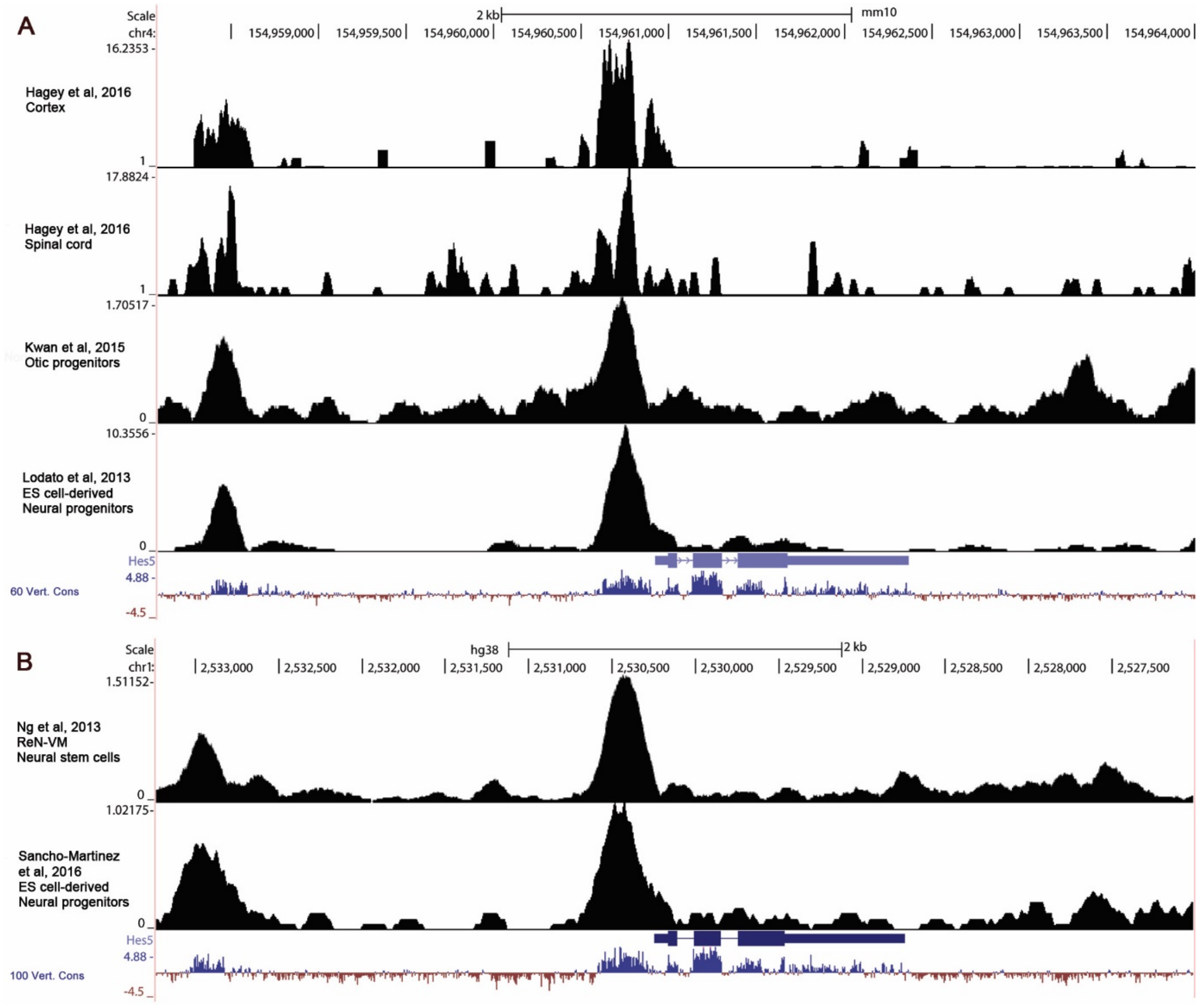

Fig. S6. Sox2 binds to the *Hes5* promoter in neural progenitors.

(A, B) Sox2 ChIP-Seq read coverage at the *Hes5* locus reveals a peak of Sox2 binding centred at ~175bp upstream of the transcription start position. Coverage tracks were obtained from i) the Cistrome project database at http://cistrome.org/db/#/, or ii) by mapping of raw reads, and visualized in the UCSC genome browser. Similar peaks were observed in alternative replicates. Sequence conservation scores are indicated at the bottom for each species.

(A) Data from mouse E11 cortical progenitors (acc. number SRR151472), E11 spinal cord progenitors (SRR151475), transformed otic progenitors (SRR1616842) and forebrain-like ES cell-derived neural progenitors (SRR630003). (B) Data from a human neural stem cell line of ventral midbrain origin (ReN-VM cells, SRR945976) and forebrain-like ES cell-derived neural progenitors (SRR1929985).

Development • Supplementary information

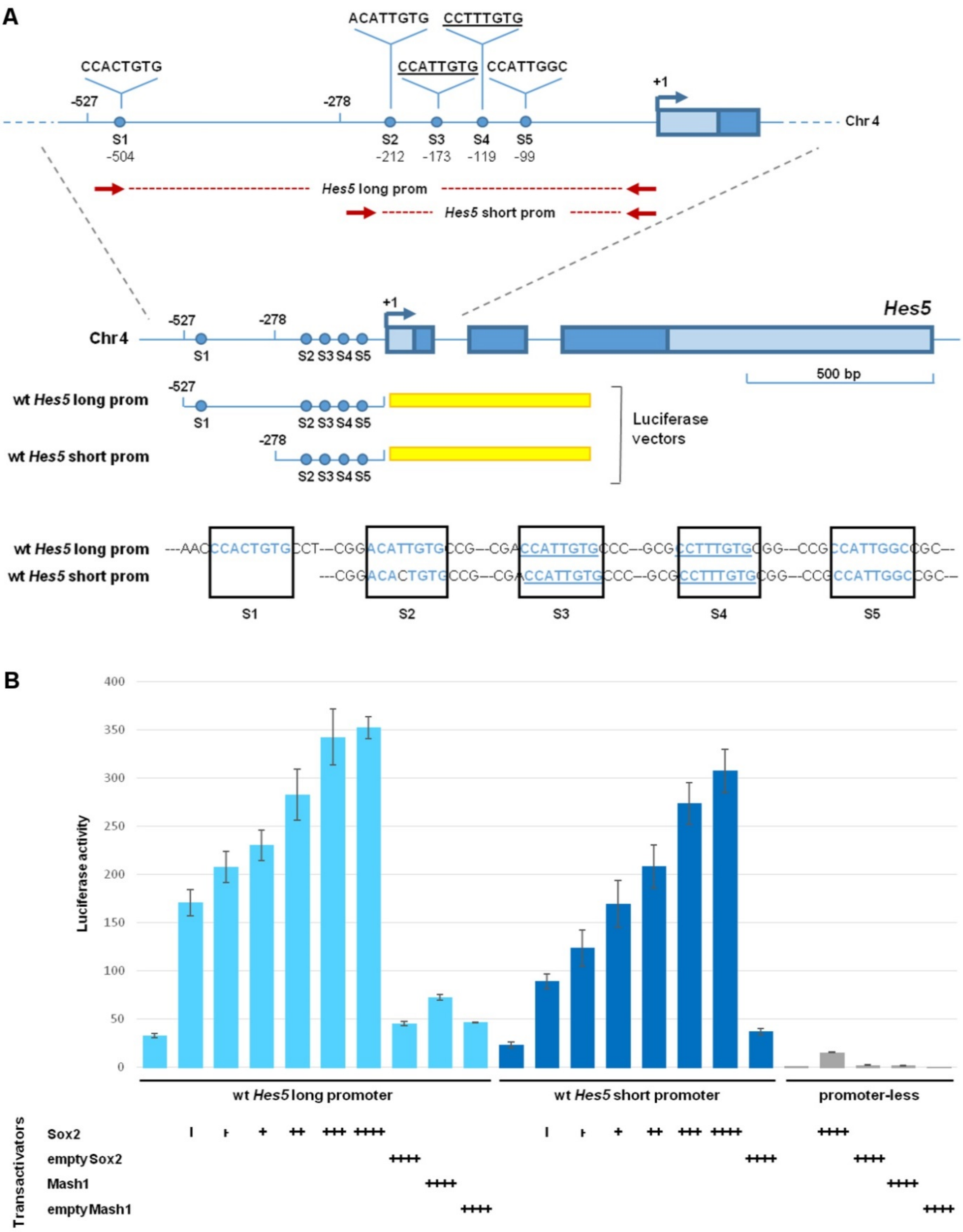

Fig. S7. Similar cis-regulatory activation of the long versus short *Hes5* promoter by Sox2.

(A) Representation of the *Hes5* promoter region. The red dotted lines indicate the position of the long (527bp) and short (278bp) regions cloned in a luciferase vector. Putative Sox2 binding sites (S1 to S5) are indicated by blue dots. The underlined sequences are the most confident

Development • Supplementary information

Sox2 consensus motifs. The confirmed sequence of the S1 to S5 binding sites in the cloned fragment is shown at the bottom. (B) Promoter activation assay in Neuro2a cells transfected with wild-type (wt) *Hes5* long or short promoter cloned in a luciferase vector. Co-transfection of increasing amounts of a *Sox2*-expressing vector, but not a *Mash1*-expressing vector or a control empty vector, resulted in a dose-dependent increase in luciferase activity driven by the wt long (light blue bars) or short (dark blue bars) *Hes5* promoter. *Sox2*- or *Mash1*-expressing vectors did not induce luciferase activity in co-transfection with a promoter-less luciferase vector. The molar ratio compared to the luciferase vector at 1 were (+, 1:0,075; ++, 1:0,125; +++, 1:0,25; ++++, 1:0,5). Results are represented as fold-change increase in activity compared to the promoter-less luciferase vector without co-transfected Sox2, which is set = 1. Values are the mean of three independent transfections, done in triplicate or duplicate in each experiment. Error bars represent ± s.e.m.

Development • Supplementary information

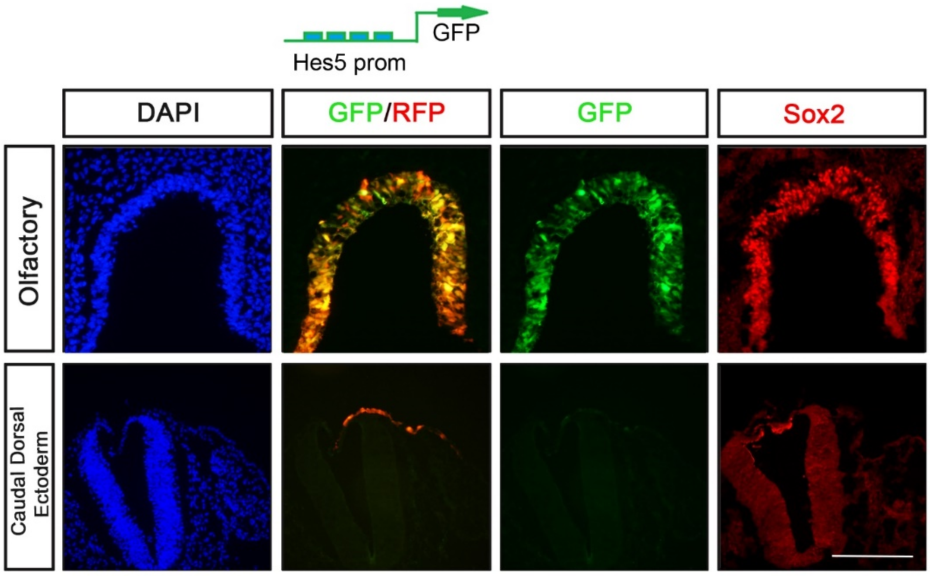

Fig. S8. *Hes5* promoter activation in olfactory epithelial cells in vivo.

In ovo electroporation of stage 9/10 chick embryos in the Sox2-positive olfactory epithelium or the Sox2-negative caudal dorsal surface ectoderm using a short *Hes5* promoter-*EGFP*-m5 construct together with a *RFP* construct and cultured to approximately stage 22. The RFP indicates the electroporation efficiency. GFP expression indicates activation of the short *Hes5* promoter in the olfactory epithelium (n=4), but not in caudal dorsal ectodermal cells (n=4), Scale bar: 100 µm.

Development • Supplementary information

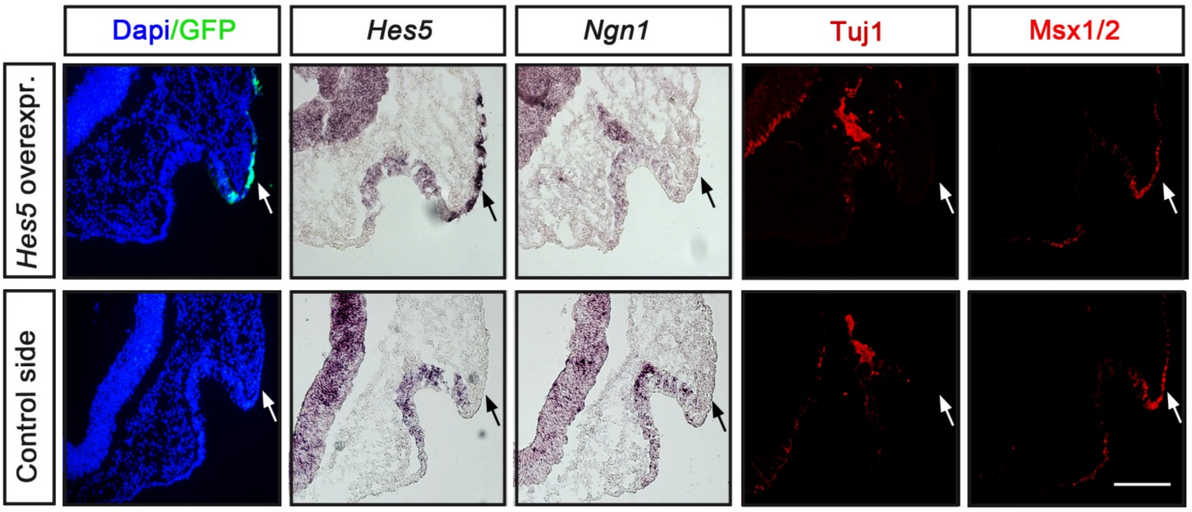

Fig. S9. *Hes5* overexpression is not sufficient to induce cells of the olfactory neuronal lineage in non-sensory head ectoderm.

Stage 10/11 chick embryos electroporated with a *GFP* vector (green) together with a *Hes5*-overexpression construct in the head ectoderm in and around the olfactory placodal region, and cultured to approximately stage 22. The electroporated *Hes5*-overexpression construct resulted in ectopic *Hes5* expression in the respiratory epithelium and head ectoderm nearby the olfactory region, but did not reduce Msx1/2 expression or induce *Ngn1* or Tuj1 expression compared to the control side (indicated by arrows) (n=5). Scale bar: 100µm.

Development • Supplementary information

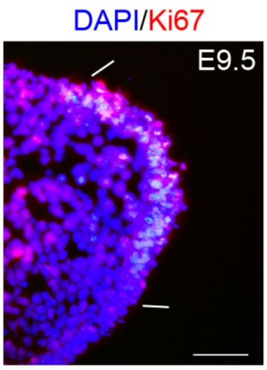

Fig. S10. The majority of olfactory placodal cells express Ki67 at E9.5

At E9.5, the majority of olfactory placodal cells express Ki67, defining proliferating cells. The olfactory placode region is indicated by white lines. Scale bar: 100µm.
